# Immobilization of Thrombin on Agarose-Based Supports for Affinity Tag Removal

**DOI:** 10.1021/acs.biomac.5c01010

**Published:** 2025-07-01

**Authors:** Juan Cruz Almada, Miguel Marco-Martin, David Roura-Padrosa, Susana Velasco-Lozano

**Affiliations:** a Instituto de Síntesis Química y Catálisis Homogénea (ISQCH), 16765 CSIC-Universidad de Zaragoza, C/Pedro Cerbuna, 12, 50009 Zaragoza, Spain; b inSEIT AG, Gesellschaftsstrasse 42, 3012 Bern, Switzerland; c Aragonese Foundation for Research and Development (ARAID), Av. Ranillas 1-D, 50018, Zaragoza, Spain

## Abstract

Thrombin, a specific serine protease, is essential in recombinant protein purification by removing affinity tags. However, its soluble form has drawbacks like instability, contamination, and limited reusability. This study explores the covalent immobilization of bovine thrombin to enhance its performance as a reusable biocatalyst. Using the CapiPy tool, surface residues suitable for immobilization on agarose supports were identified. Thrombin immobilized on glyoxyl-activated agarose showed optimal results, efficiently removing 6xHis-tags from recombinant proteins with activity comparable to the soluble enzyme. It also cleaved other peptide tags, underscoring its versatility. It retained full activity after 1.5 h at 50 °C, while the soluble form was almost inactivated. The immobilized enzyme maintained consistent performance over 10 batch cycles and achieved a space-time yield of 4.7 g·L^–1^·h^–1^. These findings highlight the potential of immobilized thrombin as a robust and cost-effective tool to improving recombinant protein purification workflows, with significant implications for both industrial and research applications.

## Graphical abstract

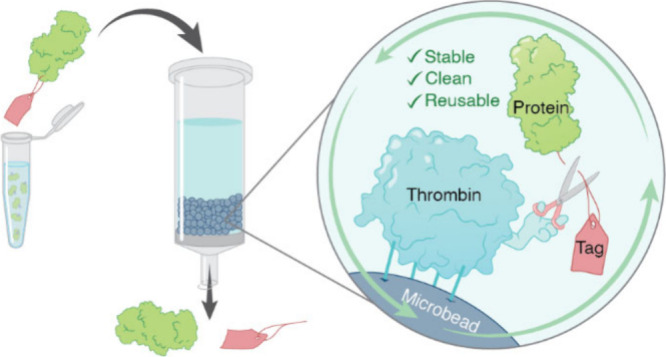

## Introduction

Recombinant protein technology has emerged as a cornerstone of modern biotechnology, with applications spanning research, medicine, and industry.^1^ Over the past few decades, numerous fusion tags have been created for recombinant protein production, serving a variety of purposes and applications.^2^ A widely used method to streamline the purification of recombinant proteins in both bacterial and eukaryotic systems involves the incorporation of affinity tags, which aid in distinguishing the target protein from the complex mixture of cellular proteins.^3^ One of the most common affinity tags is the hexa histidine (6xHis)-tag which facilitates protein purification through immobilized metal affinity chromatography (IMAC), providing a rapid and effective method to isolate the target protein from complex mixtures.^4^

While effective, the presence of affinity tags may interfere with protein functionality,^4,5^ stability,^6^ or downstream applications, necessitating their removal after purification. Methods, including natural proteolytic enzymes like thrombin, enterokinase, and tobacco etch virus (TEV) protease are frequently employed for this purpose, offering unparalleled specificity, making them widely applicable reagents for this task.^7^ Among them, thrombin, a trypsin-like serine protease, is extensively employed for cleaving peptide tags,^8^ such as the 6xHis-tag and other short peptide tags (as Strep- and FLAG-tags)^9^ engineered with a specific thrombin recognition site (typically LVPR↓GS, where thrombin cleaves at the arrow).^10^

Advantages of thrombin include its high specificity, which minimizes off-target cleavages, and its ability to operate effectively under mild reaction conditions that preserve protein stability.^7^ Additionally, thrombin has a track record of reliable performance in protein purification workflows retaining its activity in various buffer conditions.^11^ These attributes make thrombin a popular choice for applications requiring a high degree of precision and care in maintaining the integrity of recombinant proteins.

However, there are also some disadvantages when using thrombin in its soluble form for tag removal. One major issue is the potential for residual thrombin contamination in the final protein product, which can interfere with downstream applications and analyses. Free thrombin in solution also has limited reusability, which can increase costs, especially in large-scale or industrial applications. Additionally, while thrombin is generally specific, nonspecific cleavages can occasionally occur, particularly under suboptimal conditions or with prolonged exposure, leading to unintended modifications of the target protein.^12^

In response to these limitations, immobilized biocatalysts, where thrombin is immobilized on a solid support, offer an efficient and reusable solution for the selective cleavage of the affinity tag. Immobilized thrombin allows for enhanced control over the reaction, reduces the risk of contaminating the final product with free protease, and facilitates enzyme recycling, thus improving both the efficiency and sustainability of the process. Although immobilized thrombin alternatives are commercially available, such as biotinylated thrombin adsorbed onto avidin- or streptavidin-activated supports (Novagen, Madison, WI) and covalently immobilized thrombin (Sigma-Aldrich), their elevated market price restricts their routine affordability and application in large-scale processes.

Nonetheless, detailed studies exploring thrombin’s efficacy across various immobilization supports for the purpose of affinity tag elimination are notably lacking in literature. Moreover, the seldom works reporting immobilized thrombin derivatives mainly attempt to study its catalytic mechanism in fibrinogen digestion, where thrombin has been immobilized on cyanogen bromide agarose,^13^ on *p*-chlorobenzylamido agarose by hydrophobic adsorption,^14^ and on glass support bearing an active ester of *N*-hydroxysuccinimide.^15^ Other works have immobilize thrombin on polymetacrylated grafted PET fibers to assess its clotting capability for arresting bleeding in biomedical applications,^16^ or attempted to immobilize the protease on Eupergit C polyacrylamide supports to investigate the activation of protein C for antithrombotic therapy development.^17^

In this work, we aimed to enhance the performance and economic viability of thrombin as a robust heterogeneous biocatalyst for affinity tag removal (Figure 1). To achieve this, we immobilized a commercially available thrombin from bovine serum and evaluated its performance when covalently attached onto agarose-based supports. We functionalized these supports with different chemical groups to investigate their effectiveness in facilitating thrombin cleavage of a 6xHis-tag fused to a model recombinant enzyme, as well as other multimeric enzymes and affinity tags. Furthermore, in assessing its sustainability, we examined the thermal stability, reusability, scalability and economic viability of the immobilized thrombin biocatalysts.

**Figure 1:**
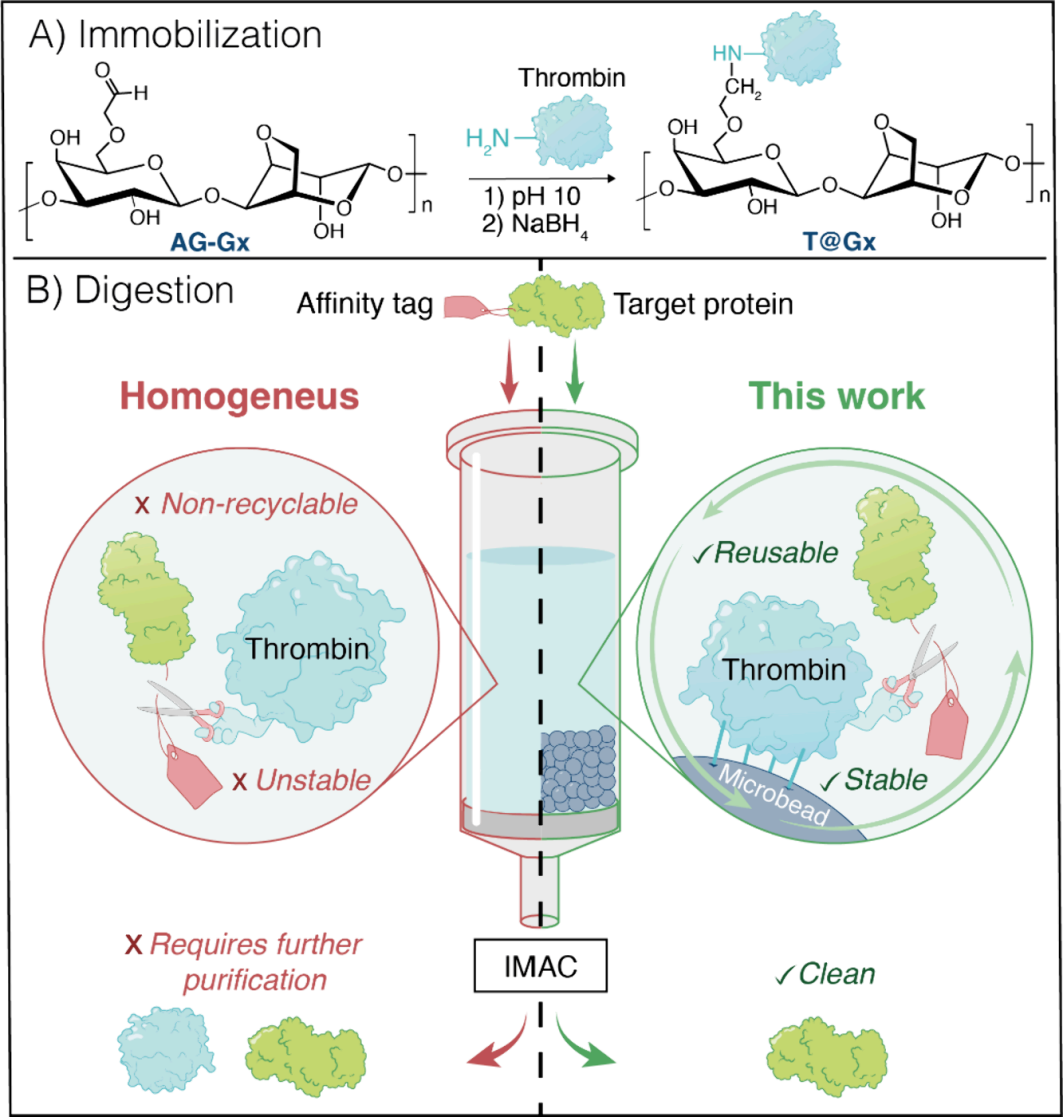
Thrombin immobilization and its application in heterogeneous affinity tag digestion. (A) Covalent immobilization on glyoxyl-agarose microbeads (AG-Gx). (B) Advantages of heterogeneous affinity tag digestion by immobilized thrombin.

## Experimental Methods

### Materials

Thrombin from bovine (catalog numbers 605157 and T7326), Boc-Val-Pro-Arg-7-amido-4-methylcoumarin hydrochloride (Boc-VPR-AMC), ethylenediamine (EDA), sodium periodate, cobalt chloride hexahydrate, and kanamycin sulfate from *Streptomyces kanamyceticus*, epichlorohydrin, sodium borohydride, glycidol, sodium periodate, SYPRO Orange, Thrombin Cleavage Capture Kit (catalog 69022, Novagen), were obtained from Sigma-Aldrich (St. Louis, MO, USA). 7-Amino-4-methylcumarin (AMC), glutaraldehyde 50% aqueous solution, iminodiacetic acid (IDA), isopropyl-β-D-thiogalactopyranoside (IPTG) was purchased from Fisher Scientific (Spain). Luria–Bertani (LB) broth and NZY autoinduction LB medium were sourced from NZYtech (Lisbon, Portugal). Agarose microbeads 4% and 6% BCL (particle size: 50–150 μm, with pore sizes of approximately 300 and 200 nm, respectively), was sourced from ABT Technologies (Madrid, Spain). Precision Plus Protein standards, Micro Bio-Spin chromatographic columns, and Bradford reagent were obtained from Bio-Rad. All other reagents and solvents were of analytical grade or higher.

### Cloning of 6xHis-, Strep-Tagged, and SpyCatcher-Fused Enzymes

The genes encoding ferredoxin-NADP^+^ reductase (FNR) from *Anabaena* sp. (as described by Medina et al.^18^), reductive aminase (RedAm) from *Rhodococcus erythropolis* (as described by Jongkind et al.^19^), and formate dehydrogenase (FDH) from *Candida boidinii* (as described by Guo et al.^20^), were codon-optimized for *E. coli* expression and synthesized by Genscript Biotech (Piscataway, NJ, USA).

The RedAm gene was fused at its N-terminus to a Strep-tag (Strep-RedAm), and the FDH gene to a SpyCatcher domain (SpyC-FDH),^21^ with a thrombin cleavage site inserted between each affinity tag and the corresponding enzyme to allow tag removal. Both constructs also feature an N-terminal 6xHis-tag upstream of the Strep-tag or SpyCatcher domain to enable IMAC purification. The codon-optimized genes encoding FNR and Strep-RedAm were then cloned into the pET28a­(+) vector using NdeI and XhoI restriction sites, whereas the SpyC-FDH construct was cloned into the pET21a­(+) vector using the same restriction sites. Standard molecular biology techniques^22^ were employed for DNA isolation, plasmid purification, and DNA sequencing to confirm the correct insertion and sequence integrity of the gene.

### Bacterial Strains and Growth Conditions

N-terminal 6xHis-tagged enzymes and proteins including ferredoxin NADPH reductase (FNR) from *Anabaena* sp., green fluorescent protein (GFP) from *Aequorea victoria*,^23^ formate dehydrogenase (FDH) from *Candida boidinii*,^24^ l-alanine dehydrogenase (AlaDH) from *Bacillus subtilis*,^24^ and glycerol dehydrogenase (GlyDH) from *Geobacillus stearothermophilus*,^25^ were cloned and overexpressed in competent *E. coli* BL21­(DE3) cells. Additionally, a Strep-tagged reductive aminase from *Rhodococcus erythropolis* (Strep-RedAm) and an FDH construct fused to a SpyCatcher domain (SpyC-FDH) were also expressed in the same host strain. Competent cells were transformed with the respective plasmid, following previously established protocols. Briefly, 1 mL of an overnight culture of *E. coli* BL21­(DE3) containing the plasmid was used to inoculate 50 mL of Luria–Bertani (LB) medium supplemented with kanamycin (final concentration 30 μg·mL^–1^). Exceptions included FNR, which was cultured in NZY autoinduction medium; Strep-RedAm, which was cultured in Terrific Broth (TB); and SpyC-FDH, which was cultivated in LB medium supplemented with ampicillin (final concentration 50 μg·mL^–1^). The cultures were incubated aerobically at 37 °C with orbital shaking at 250 rpm until an OD_600_ of 0.6 was reached. Induction was initiated with 1 mM IPTG, except for FNR, which did not require IPTG addition and Strep-RedAm, which was induced with 0.5 mM IPTG. Enzyme expression times were as follows: GFP and AlaDH for 3 h at 37 °C; FDH, GlyDH, Strep-RedAm and SpyC-FDH for 18 h at 21 °C; and FNR autoinduced for 17 h at 37 °C.

Following induction, cells were harvested by centrifugation at 4,211 × g for 30 min at 4 °C. The supernatant was discarded, and the cell pellet was resuspended in 5 mL of the respective enzyme buffer supplemented with 10 mM imidazole: GFP, FDH, GlyDH, and SpyC-FDH used 25 mM sodium phosphate buffer at pH 7.0; AlaDH used 25 mM potassium phosphate buffer at pH 7.0; and FNR and Strep-RedAm used 100 mM Tris buffer at pH 8.0. Cells were lysed by sonication using a Sonopuls 4050 ultrasonic homogenizer (Bandelin) at 30% amplitude (5 s ON/5 s OFF) for 20 min at 4 °C. The suspension was then centrifuged at 10,528 × g for 30 min at 4 °C, and the pellet was discarded. The resulting supernatant, containing cell extracts with the 6xHis-tagged protein, was collected for subsequent purification and 6xHis-tag digestion.

### Purification of Recombinant Produced Enzymes and Proteins by IMAC

To purify each 6xHis-tagged enzyme, 10 volumes of the crude cell extract (derived from a 50 mL cell pellet resuspended in the appropriate buffer) were combined with 1 volume of cobalt-activated agarose microbeads (AG-Co^2+^) and incubated with orbital shaking for 1 h at 4 °C. Following incubation, the suspension was filtered, and the enzyme-bound microbeads were washed with 5 volumes of the corresponding enzyme buffer. The enzyme was then eluted by adding 6 volumes of 300 mM imidazole in the appropriate buffer and incubating for 1 h at 4 °C with orbital shaking. After elution, the protein was diafiltrated using a tangential ultrafiltration unit with a 10 kDa cutoff to remove residual imidazole. SDS-PAGE and Bradford protein assays were conducted after each production step to assess the enzyme’s purity, concentration, and specific activity.

### Protein Quantitation

Protein concentration was measured using the Bradford method.^26^ Briefly, 5 μL of each sample (concentration range: 0.2–0.9 mg·mL^–1^) was added to a well in a clear 96-well microplate, followed by the addition of 200 μL of Bradford reagent (Bio-Rad) diluted 1:4 in distilled water. The mixture was incubated for 10 min at room temperature. Following incubation, absorbance was measured at 595 nm using an Epoch 2 Microplate Spectrophotometer (BioTek) with Gen5 software. Protein concentration was determined by interpolating the absorbance values against a standard curve generated with commercial BSA standards (Thermo Fisher).

### Thrombin Activity by Spectrofluorometric Assay

Thrombin activity was measured by quantifying the release of free fluorescent 7-amino-4-methylcoumarin (AMC), which has excitation and emission wavelengths of 380 and 450 nm, respectively, following the hydrolysis of the fluorogenic peptide synthetic substrate Boc-VPR-AMC (Scheme S1). Briefly, 200 μL of a reaction mixture containing 5 μM Boc-VPR-AMC in 50 mM Tris buffer pH 8.0 was prepared and added to a well in a black 96-well microplate. The reaction was initiated by adding 5 to 20 μL of appropriately diluted enzymatic solution or suspension and incubated at 30 °C. The increase in fluorescence was recorded using a Synergy H1Multimode Reader (BioTek) with Gen6 software. One unit of thrombin activity was defined as the enzyme amount needed to produce 1 μmol of AMC per minute under these conditions.

### Preparation of Glyoxyl-Activated Agarose (AG-Gx)

To prepare glyoxyl-activated agarose (AG-Gx) we followed a previous reported methodology.^27^ Briefly, 105 g of agarose 4% BCL were suspended in 80 mL of water containing 3.4 g NaOH and 1.4 g NaBH_4_ at 4 °C. Subsequently, 36 mL of glycidol was gradually added to the suspension under mild continuous stirring. The reaction mixture was then stirred gently using a stainless steel propeller for 16 h at room temperature. Afterward, the agarose was thoroughly washed with an excess of water to remove any unreacted materials. The washed agarose was then treated with 10 volumes of 0.1 M sodium periodate solution in water, stirring for 2 h at room temperature to complete the activation process. The degree of functionalization of AG-Gx (100 μmol·g^–1^) was confirmed through measuring the remaining NaIO_4_, as previously described.^27^

### Preparation of Epoxy-Activated Agarose (AG-E)

To prepare epoxy-activated agarose (AG-E) we employed a previously reported method.^28^ Briefly, 105 g of agarose 4% BLC were suspended in a mixture of 440 mL water, 160 mL acetone, 32.8 g NaOH, and 2 g NaBH_4_ at 4 °C. Next, 110 mL of epichlorohydrin was gradually added to the suspension under gentle, continuous stirring. The reaction mixture was then stirred with a stainless steel propeller for 16 h at room temperature. Following this, the agarose was thoroughly washed with water to remove any residual reactants and stored at 4 °C for future use. The functionalization degree of AG-E (38 μmol·g^–1^) was verified by iodometric analysis, as previously described.^29^

### Preparation of Amino-glutaraldehyde-Activated Agarose (AG-A/G)

Based on a previously established procedure, we prepared amino-glutaraldehyde-activated agarose (AG-A/G).^30^ Briefly, 10 g of AG-E resin were suspended in 10 volumes of a 2 M ethylenediamine (EDA) water solution, adjusted to pH 11. The mixture was stirred using gentle rotation for 16 h at room temperature to facilitate the amination reaction. Afterward, the agarose was thoroughly washed with an excess of water to remove any unreacted EDA. The amine-functionalized agarose (AG-A) was then combined with 10 volumes of a 15% glutaraldehyde solution in 200 mM sodium phosphate buffer, pH 7. This reaction mixture was gently stirred with gentle rotation for 16 h at room temperature, shielded from light to prevent glutaraldehyde degradation. After the reaction, the agarose was washed extensively with water to eliminate any remaining glutaraldehyde. The resulting AG-A/G was used immediately after glutaraldehyde activation. Aldehyde functionalization with glutaraldehyde (∼38 μmol·g^–1^) was confirmed by titration with Schiff’s reagent as previously reported.^30^

### Preparation of Cobalt-Activated Agarose Microbeads (AG-Co^2+^)

To prepare cobalt-activated agarose microbeads 6% BCL (AG-Co^2+^), 10 g of AG-E resin were mixed with 100 mL of 0.5 M iminodiacetic acid (IDA) solution, adjusted to pH 11.0. The mixture was stirred with a stainless steel propeller at room temperature and allowed to incubate overnight. Afterward, the IDA-functionalized agarose (AG-IDA) was thoroughly washed with water to remove any unreacted IDA. To hydrolyze any residual epoxy groups, AG-IDA was then treated with 100 mL of 0.5 M H_2_SO_4_ and incubated at room temperature for 2 h, followed by extensive washing with deionized water. To introduce the metal group, the AG-IDA was subsequently mixed with 100 mL of cobalt chloride solution (30 mg·mL^–1^) and incubated under stirring for 1 h at room temperature with a stainless steel propeller. The resulting AG-Co^2+^ was thoroughly washed with excess water to eliminate any remaining cobalt chloride and stored at 4 °C for future applications.

### Thrombin Immobilization. Thrombin Pretreatment Prior Immobilization

For this study, we employed a commercially available thrombin from bovine (cat. 605157, from Sigma-Aldrich) exhibiting higher purity than another available one (cat. T7326 from the same supplier) (Figure S1). Reconstituted commercial thrombin was buffer-exchanged into 25 mM Tris buffer at pH 7.0 using a tangential flow filtration system (Amicon Ultra-15) equipped with a 10 kDa molecular weight cutoff membrane, effectively removing the excipients.

### Immobilization on AG-Gx

To prepare immobilized thrombin on AG-Gx (T@Gx and T_HL_@Gx), 100 mg of AG-Gx resin was mixed with 1 mL of thrombin solution (0.01 or 0.1 mg·mL^–1^ for T@Gx or T_HL_@Gx, respectively) in 100 mM sodium bicarbonate buffer at pH 10. The mixture was incubated under orbital shaking for 2 h at 4 °C to facilitate enzyme immobilization. Following this incubation, the preparation was treated with a 1 mg·mL^–1^ sodium borohydride solution in 100 mM sodium bicarbonate buffer, pH 10, to reduce the formed Schiff bases, stabilizing the immobilized enzyme through irreversible amide bond formation. The resulting immobilized thrombin was filtered and thoroughly washed with 150 mM NaCl in 25 mM Tris buffer pH 8.0, to remove unbound components. Finally, the immobilized biocatalyst was stored at 4 °C for further use.

### Immobilization on AG-E

To prepare immobilized thrombin on AG-E (T@E), 100 mg of AG-E resin was combined with 1 mL of thrombin solution (0.01 mg·mL^–1^) in 50 mM sodium phosphate buffer, pH 8. The mixture was incubated with orbital shaking at 4 °C for up to 5 h to promote enzyme immobilization. After incubation, the preparation was filtered and then incubated overnight at 4 °C in 10 volumes of 1 M glycine solution to block any remaining free epoxy groups. The immobilized thrombin was subsequently filtered and thoroughly washed with 150 mM NaCl in 25 mM Tris buffer pH 8.0 to ensure complete removal of unreacted glycine. Finally, the immobilized biocatalyst was stored at 4 °C for future applications.

### Immobilization on AG-A/G

To prepare the AG-A/G-immobilized thrombin (T@A/G), 100 mg of AG-A/G resin freshly prepared was combined with 1 mL of thrombin solution (0.01 mg·mL^–1^) in 200 mM sodium phosphate buffer at pH 7 and incubated at 4 °C with orbital shaking for 2 h to support enzyme immobilization. Following incubation, the mixture was filtered and then incubated overnight at 4 °C with 10 volumes of 1 M glycine solution to block unreacted aldehyde groups. The resulting immobilized thrombin was filtered again and rinsed thoroughly with 150 mM NaCl in 25 mM Tris buffer pH 8.0 to clear any remaining glycine. Finally, the immobilized biocatalyst was stored at 4 °C for later applications.

### Thermal Stability of Thrombin Biocatalysts

Soluble or immobilized thrombin was placed in a 0.2 mL Eppendorf tube and prepared by dissolving in 50 mM Tris buffer pH 8.0, at concentrations of 0.022–0.4 mg·mL^–1^ for soluble thrombin, while immobilized thrombin was prepared as a 1:2.5 resin-to-buffer suspension. The samples were incubated at 50 °C in a thermoblock for 0.5 or 1.5 h. Following incubation, the samples were immediately cooled in an ice bath for 10 min. Residual thrombin activity was then measured using the spectrofluorometric assay.

### Affinity Tag Removal

Soluble or immobilized thrombin was introduced into a 1.2 mL Bio-Spin chromatography column, followed by the addition of a 6xHis-tagged enzyme/protein in 50 mM Tris buffer pH 8.0, achieving a final volume of 250 μL. The final concentrations of thrombin and the target enzyme/protein were 2 μg·mL^–1^ and 200 μg·mL^–1^, respectively, corresponding to a thrombin-to-enzyme mass ratio of 1:100. The mixture was incubated at 4 °C with gentle mixing for various time intervals (1, 2, 3, 4, or 20 h). Following incubation, the digested enzyme/protein was collected by filtration (eluate 1) and transferred to a second 1.2 mL Bio-Spin chromatography column preloaded with 40 mg of AG-Co^2+^ resin. This step was used to separate the cleaved 6xHis-tag and undigested 6xHis-tagged enzyme/protein from the digested enzyme/protein. The mixture was incubated for 1 h at 4 °C with gentle mixing. The digested enzyme/protein present in the flow-through was then collected (eluate 2) for protein content analysis.

### Recyclability of Immobilized Thrombin

To perform each His-tag digestion cycle, 5 mg of immobilized thrombin on AG-Gx loaded at 1 mg·g_support_^–1^ (T_HL_@Gx) were placed in a 1.2 mL Bio-Spin chromatography column and mixed with 250 μL of 6xHis-GFP (2 mg·mL^–1^). The mixture was incubated at 4 °C with gentle mixing. After incubation, the GFP solution (eluate 1) was collected by filtration and transferred to another 1.2 mL Bio-Spin chromatography column preloaded with 40 mg of AG-Co^2+^ resin. This mixture was incubated for 1 h at 4 °C with gentle mixing to separate the cleaved 6xHis-tag and undigested 6xHis-GFP from the digested GFP. The flow-through containing the digested GFP (eluate 2) was collected for protein content analysis. Following each digestion cycle, the immobilized thrombin was washed once with 10 volumes (50 μL) of 25 mM Tris buffer pH 7.0 supplemented with 150 mM NaCl before starting the next digestion cycle.

### Affinity Tag Digestion Intensification

To assess the scalability of the heterogeneous thrombin, we adapted the previously described protocol for affinity tag removal, using 6xHis-GFP as the target protein and T_HL_@Gx as the biocatalyst. The digestion was performed at various thrombin-to-GFP mass ratios (1:50, 1:100, 1:200, and 1:500) to evaluate the efficiency and scalability of the process.

### Storage Stability of Immobilized Thrombin

Immobilized thrombin on AG-Gx (T@Gx) was prepared, and excess buffer was removed by centrifugation at 1000 rpm for 30 s. The biocatalyst was then stored at 4 °C. After 30 and 90 days, the residual activity of the biocatalyst was assessed using the spectrofluorometric assay.

### Thermal Shift Assay

Soluble thrombin (10 μM, 40 μL) was mixed with SPYRO Orange dye (10 μL of a 50-fold dilution in DMSO from the commercial one) in 50 mM Tris buffer pH 8.0. The mixture was subjected to thermal denaturation using a QuantStudio 5 real-time PCR thermal cycler (ThermoFisher) with a temperature program consisting of an initial hold at 25 °C for 2 min, followed by a linear temperature increase at a rate of 1 °C·min^–1^ until reaching 95 °C, where it was held for 2 min. Fluorescence data were recorded continuously during the thermal ramp, and analysis was performed using QuantStudio Design & Analysis software version 1.3.1. The melting temperature (*T*_m_) was determined according to a nonlinear Boltzmann fitting of the fluorescence emission data, or an analysis of the first derivative of fluorescence emission with respect to temperature.^31^

### Intrinsic Tryptophan Fluorescence Assay

A volume of 100 μL of either soluble or immobilized thrombin (0.035 mg·mL^–1^) in 50 mM Tris buffer (pH 8.0) was placed in a 96-well black microplate. Fluorescence emission spectra were recorded between 310 and 450 nm following excitation at 280 nm, using an emission bandwidth of 2 nm. The resulting spectra were blank-corrected using their respective buffer and immobilization support controls, then normalized to their maximum fluorescence intensity. Subsequently, the spectra were fitted to a Gaussian function using Origin 2020b software to determine the maximum emission wavelength.

### Computational Methods

CapiPy^32^ package was used to perform the surface analysis of Thrombin. All analyses were performed using a modified version of the 3D structure with PDB ID 3PMB. The following modifications were made to the initial PDB file: the light chain was left unchanged, maintaining its original numbering; the heavy chain numbering was adjusted to range from 1 to 237, and the missing loops were modeled using MODELER. The complete sequence of the PDB file is provided in the Supporting Information (Figure S2).

Surface and cluster analysis were performed using CapiPy’s module.^33^ Flexibility analysis was performed using the MARTINI force field. Protein structures were transformed using the martinize2 functionality and simulated with the Martini^34^ force field in GROMACS.^35^ Briefly, after the protein was martinized, it was solvated in explicit water, and the system was energy-minimized. Following successful equilibration (300 K, 100 fs), a 5 ns production run was carried out. Flexibility was quantified as the average RMSF of each residue.

## Results and Discussion

### Thrombin Immobilization

Thrombin is a globular protein belonging to the serine protease family, consisting of two polypeptide chains: a heavy chain (∼33 kDa) and a light chain (∼5 kDa), which are covalently linked via a disulfide bond. The catalytic site of bovine thrombin is located within the heavy chain and contains the classic serine protease triad—Ser, His, and Asp—responsible for its enzymatic activity (Figure 2A). To determine the most effective immobilization strategy, we conducted an analysis of its surface properties. Thrombin possesses numerous lysine residues exposed on its surface (Figure S3A), making lysine the second most abundant residue (Figure 2B). This abundance makes thrombin an excellent candidate for immobilization through amino-reactive functional groups such as epoxides and aldehydes.^28,36^ To identify the surface-exposed lysine residues most prone to lead thrombin immobilization through these groups, we analyzed their solvent-accessible surface area (SASA), surface exposure measured through their HSEβ^37^ and cluster presence (defined as three or more lysine where their Cβ is at less than 5Å distance) using the Python-based application CapiPy.^32^ Using these criteria, we identified two potential sites for immobilization. The first cluster consists of K60, K81 and K83, while the second cluster includes K100, K215, K216 and K220 (Figure 2A). Note that all residue numbers correspond to the modified PDB version (see Materials and Methods, and Figure S2 for further details). The residues forming these two lysine clusters are close and well exposed to the solvent (Figures 2A and S3B). Notably, cluster 1 is located in a highly flexible region of the protein, while cluster 2 resides in a less flexible area (Figures 2C and S3B). Flexible regions are often targeted for enzyme immobilization, as they are generally more adaptable and less likely to interfere with the enzyme’s active site or overall structure, thus helping to preserve its functional integrity.^38^ Furthermore, the potential orientation of immobilized thrombin via these clusters minimally hinders substrate access since are located on a plane backward the catalytic triad (Figure 2A).

**Figure 2:**
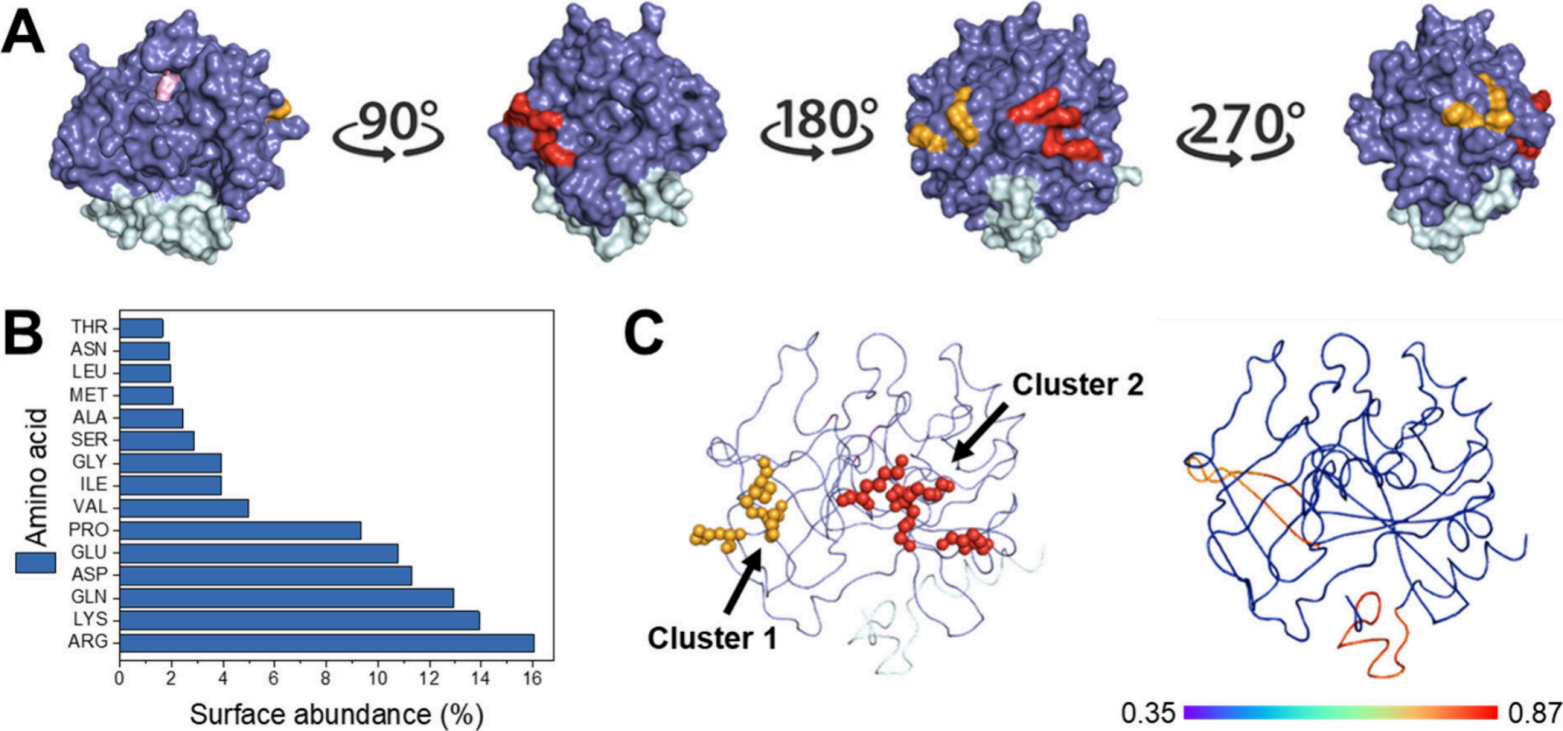
Structural analysis of thrombin (PDB 3PMB). (A) Surface-exposed lysine clusters. The heavy chain is depicted in slate blue, and the light chain in pale cyan. The catalytic triad residues (S173, H29, D76) are marked in pink. Cluster 1 (K60, K81, K83) is highlighted in orange, while Cluster 2 (K100, K215, K216, K220) is shown in red. (B) Amino acid surface abundance. (C) Flexibility analysis. Left: Cluster localization is depicted with orange spheres representing cluster 1, and red spheres representing cluster 2. Right: Flexibility results from a 5 ns molecular dynamics (MD) simulation, with warmer colors (red) highlighting regions of higher flexibility and colder colors (blue) indicating more rigid areas. The numbers indicate the average RMSF. Note that all residue numbers correspond to the modified PDB version (see Experimental Methods and Figure S2 for further details).

After identifying promising lysine clusters as candidates for effective thrombin immobilization, we selected 4% cross-linked porous agarose microbeads (AG) as the immobilization matrix. These microbeads, with an average diameter of 50–150 μm and an internal pore size of 300 nm, offer suitable porosity for the intended application. They can accommodate a monolayer of immobilized thrombin (approximately 5 nm in protein diameter), leaving approximately 290 nm of free space to facilitate the access and exit of protein targets. Subsequently, we prepared three distinct and well-characterized types of functionalized agarose microbeads designed to target lysine residues: epoxy-activated agarose (AG-E), glyoxyl-activated agarose (AG-Gx), and amino/glutaraldehyde-activated agarose (AG-A/G) (Scheme S2).

On the prepared supports, thrombin exhibits fast attachment rates achieving 100% immobilization yield (Ψ) in less than 30 min when bound to both aldehyde-functionalized agarose, (Table 1, entries 2 and 3; Figure S4). In contrast, AG-E reaches lower immobilization yield in 4-fold longer time (Table 1, entry 1; Figure S4). Although thrombin is rapidly attached on aldehyde-activated supports (AG-Gx and AG-A/G), we maintain the incubation with the support up to 2 more hours allowing the formation of multipoint attachment,^39^ thus providing higher stabilization to the immobilized thrombin.^40,41^ Beyond the immobilization yield, the heterogeneous biocatalysts displayed different recovered activity depending on the used support as detailed below.

**Table 1:** Immobilization Parameters of Thrombin Immobilized on Functionalized Agarose Microbeads

			Enzyme load (mg·g_support_^–1^)			
Entry	Abbreviation	Support	Offered	Loaded	Ψ^a^(%)	Recovered specific activity^b^ (%)
1	T@E	AG-E	0.1	0.059	59	60 ± 6
2	T@Gx	AG-Gx	0.1	0.1	100	75 ± 6
3	T@A/G	AG-A/G	0.1	0.1	100	41 ± 2
4	T_HL_@Gx	AG-Gx	1.0	1.0	99	113 ± 3

a Immobilization yield (Ψ) is calculated as (immobilized activity/offered activity) × 100.

b Recovered activity (%) represents the ratio of the specific activity of the immobilized enzyme to that of the soluble enzyme. The specific activities of the thrombin biocatalysts were measured by assessing the digestion of a 6xHis-FNR (0.2 mg·mL^–1^) at a thrombin-to-enzyme mass ratio of 1:100 in 50 mM Tris buffer pH 8.0, using a 1:50 w/w support-to-FNR volume ratio at 4 °C for 1 h. Under these conditions, soluble thrombin digests 62 mg_FNR_/mg_thrombin_·h, corresponding to 100% recovered specific activity.

To evaluate the residual activity of immobilized thrombin, we conducted a 6xHis-tag digestion of a model monomeric enzyme, the 6xHis-FNR, comparing the immobilized thrombin’s performance with that of its soluble counterpart. Thrombin activity was quantified by measuring the mass of 6xHis-FNR digested per mass of thrombin used within 1 h at 4 °C, maintaining a consistent thrombin-to-enzyme mass ratio of 1:100. Following thrombin digestion, the resulting enzyme mixture product is collected in what we refer to as eluate 1, comprising three components: the undigested 6xHis-enzyme, the digested enzyme, and the residual 6xHis-tag. This mixture is subsequently processed through an IMAC column to remove the undigested enzyme and residual 6xHis-tag, yielding the purified final product in what we refer to as eluate 2 (Figure 3A).

**Figure 3:**
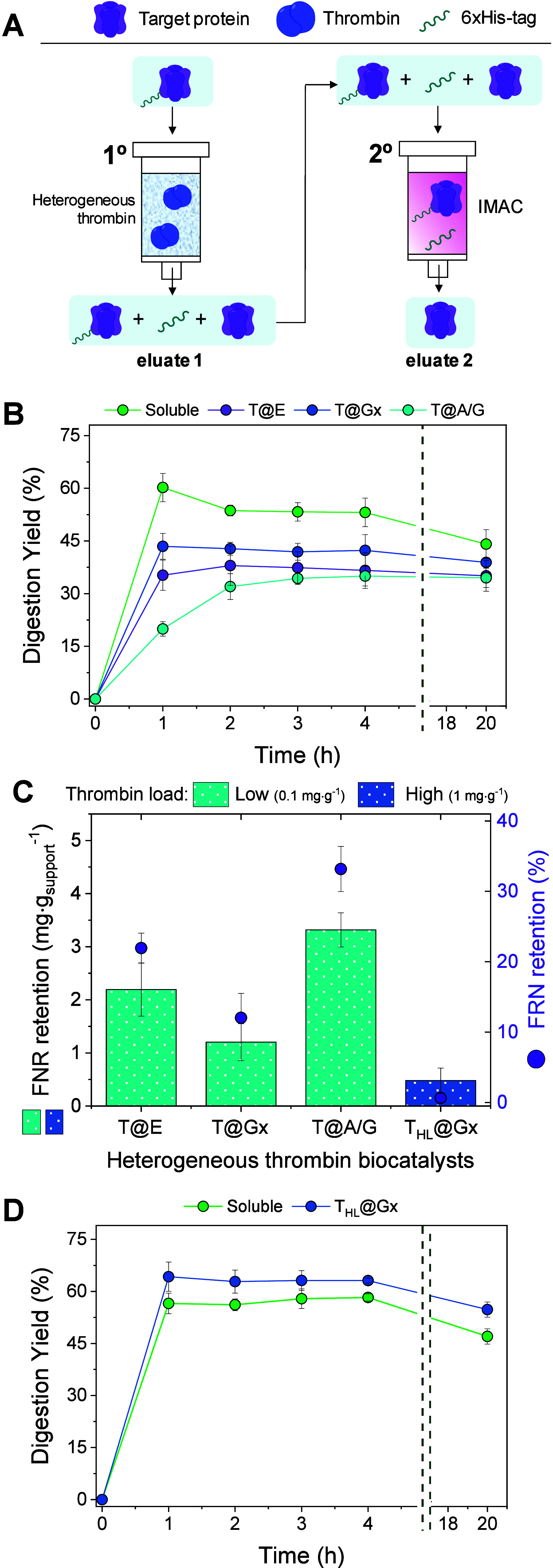
Affinity tag digestion kinetics by various heterogeneous thrombin preparations. (A) 6xHis-tag digestion workflow. (B) Digestion kinetics over time. The enzyme 6xHis-FNR (0.2 mg·mL^–1^) was incubated with thrombin biocatalysts at a target protein-to-thrombin mass ratio of 100:1 in 50 mM Tris buffer pH 8.0, using a support-to-FNR ratio of 1:50 (w/w), at 4 °C with gentle rotation at 30 rpm. (C) Nonspecific retention of FNR on different immobilized thrombin supports during 6xHis-tag digestion; low and high thrombin loads correspond to 0.1 mg and 1.0 mg of thrombin per gram of support, respectively. (D) The enzyme 6xHis-FNR (2.0 mg·mL^–1^) was incubated with either soluble thrombin or T_HL_@Gx at a target protein-to-thrombin mass ratio of 100:1 in 50 mM Tris buffer (pH 8.0), with a support-to-FNR ratio of 1:50 (w/w), at 4 °C under gentle rotation at 30 rpm.

Interestingly, the AG-Gx support produced the most active immobilized thrombin (T@Gx), retaining 75% of its original activity despite the more stringent conditions required for its preparation, including thrombin incubation at pH 10 followed by a NaBH_4_ reduction step (Table 1, entry 2). In contrast, thrombin immobilized on AG-E and AG-A/G supports (T@E and T@A/G, respectively) displayed significantly lower recovered activities, being 1.3- and 1.8-fold less active than T@Gx, respectively, despite requiring milder immobilization conditions (Table 1, entries 1 and 3). This variation is likely due to differences in thrombin orientation upon immobilization on AG-A/G, where the presence of positively charged amines may orient thrombin through its more negatively charged regions (clusters of aspartic and glutamic acid residues), leading to a less favorable binding orientation (Figure S5). Similar effects have been reported in other studies, such as thrombin immobilized on glass beads functionalized with *N*-hydroxysuccinimide ester moieties, which target aspartic and glutamic acid residues on thrombin’s surface. This strategy resulted in a biocatalyst retaining only 6% of its initial proteolytic activity. The marked reduction in activity was primarily attributed to steric hindrance limiting substrate accessibility due to suboptimal thrombin orientation, as well as the depletion of free amino groups essential for ionic interactions with the fibrinogen substrate.^15^

To better understand the residual activity of immobilized thrombin, we examined the 6xHis-tag digestion kinetics to determine the optimal digestion time to maximize the yield of digested enzyme. The yield of digested FNR was quantified as the percentage of protein concentration in eluate 2 relative to the initial target protein concentration (Figure 3A). Additionally, we analyzed both the mass of digested FNR (eluate 2) and the mass of nonspecifically adsorbed FNR retained during incubation with the immobilized thrombin biocatalyst. FNR retention was quantified as the percentage decrease in protein mass in eluate 1 relative to the initial protein concentration.

Notably, neither soluble nor immobilized thrombin achieved complete (100%) digestion of 6xHis-FNR, even after extending the incubation to 20 h (Figure 3B). Interestingly, the soluble thrombin, T@Gx and T@E biocatalysts reached their respective maximum 6xHis-tag digestion within the first hour, whereas T@A/G required up to 3 h to achieve its maximum digestion yield (Figure 3B). Furthermore, the T@Gx biocatalyst exhibited the lowest FNR retention, with values of 1.2 ± 0.3 mg·g_support_^–1^, corresponding to 12% of the initial target protein amount when digesting 0.2 mg·mL^–1^ of 6xHis-FNR. This retention was 1.8 to 2.8 times lower compared to the other two immobilized thrombin preparations (Figure 3C). The increased FNR retention observed on the AG-A/G support can be primarily attributed to the positively charged amino groups introduced by agarose functionalization with EDA, which serve as anionic protein exchangers.^30^ These groups ionically adsorb FNR, diminishing its recovery and impairing efficient digestion, as evidenced by the lowest observed digestion rates.

### Stability of Immobilized Thrombin

To ensure an immobilized thrombin preparation that is both efficient and stable, we evaluated the thermal stability of the prepared biocatalysts. First, the melting temperature of soluble thrombin was determined using a thermal shift assay, revealing a melting temperature (*T*_m_) of 50.8 °C (Figure S6). Subsequently, both soluble and immobilized samples were incubated at 50 °C to assess their thermal stability. As anticipated, soluble thrombin lost over 95% of its initial activity after 90 min under these conditions. In contrast, T@Gx emerged as the most stable immobilized preparation, retaining up to 60% of its initial activity over the same incubation period (Figure 4A). These results align with prior studies highlighting the exceptional stabilization of other enzymes through immobilization on AG-Gx supports.^36,41,42^ Consistent with our findings, enhanced thermal stability of immobilized thrombin has also been reported when using polymethacrylate-based supports activated with epoxides.^17^

**Figure 4:**
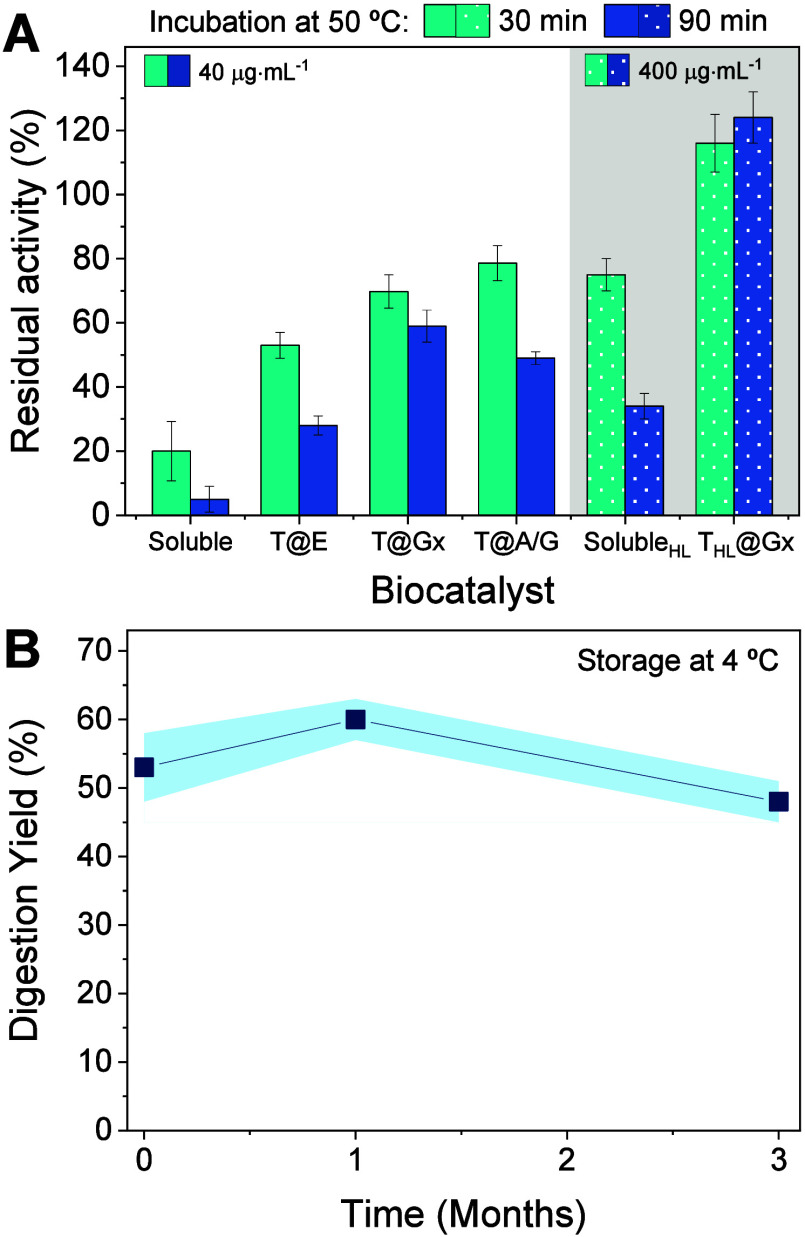
Thermal and storage stability of thrombin biocatalysts. (A) Thermal stability at 50 °C incubated in 50 mM Tris buffer pH 8.0. (B) Storage stability of T@Gx at 4 °C with the standard deviation of three independent replicates represented as a light blue shaded area.

In addition, T@Gx showed remarkable storage stability at 4 °C after 30 and 90 days stored at these conditions, preserving more than 90% of its initial activity (Figure 4B). Based on its superior performance, including high residual activity, low target protein retention, and excellent thermal and storage stability, we selected T@Gx for further analysis.

Once selecting T@Gx as the most effective heterogeneous biocatalyst, we evaluated its performance with a 10-fold increase in immobilized thrombin load (1.0 mg_thrombin_·g_support_^–1^), referred to as T_HL_@Gx. Despite the higher loading, T_HL_@Gx achieves 99% immobilization yields within the same incubation time as T@Gx (Table 1, entry 4). Remarkably, T_HL_@Gx outperforms its soluble counterpart in 6xHis-tag digestion efficiency, retaining 113% of its original activity upon immobilization (Table 1, entry 4). This enhanced performance is reflected in an average digestion yield that is 14% ± 3% higher than that achieved with the soluble thrombin over the entire digestion time window (1 to 20 h) under identical target enzyme-to-thrombin mass ratio conditions (Figure 3D). Moreover, T_HL_@Gx demonstrates significantly reduced nonspecific retention of the target 6xHis-FNR, retaining 2.5 times less protein mass per gram of heterogeneous biocatalyst compared to T@Gx (Figure 3C), corresponding to only 0.67% of the initial target enzyme concentration (2.0 mg·mL^–1^). This lower nonspecific protein retention can be attributed to the reduced availability of free surface area on the support, resulting from the higher thrombin coverage per unit area when loading 10 times more thrombin per gram of support.

To further investigate the enhanced catalytic performance of T_HL_@Gx compared to both T@Gx and soluble thrombin, we evaluated potential conformational changes induced by immobilization using tryptophan fluorescence spectroscopy. Both heterogeneous biocatalysts (T@Gx and T_HL_@Gx) showed red shifts in their emission maxima relative to the soluble enzyme, indicating structural alterations (Figure S7). The low-loading biocatalyst (T@Gx) exhibited a pronounced red shift (8.7 nm), suggesting significant conformational perturbation. In contrast, T_HL_@Gx showed a smaller shift, indicating that its native structure is better preserved, likely due to fewer enzyme–support interactions at high loading, which minimize structural distortion. Interestingly, this partial preservation of the native fold in T_HL_@Gx appears to result in a more favorably exposed catalytic triad compared to the soluble enzyme, whose conformation in solution may be more flexible but less catalytically efficient.

To complement these findings, we performed an *in silico* analysis evaluating thrombin immobilization via lysine cluster 1 or cluster 2. The results suggest that attachment via cluster 2 confers greater solvent accessibility to the catalytic site than attachment via cluster 1, as reflected by a broader opening angle of the active site (Figure S8). This suggests that at higher enzyme densities, as in T_HL_@Gx, immobilization may preferentially occur via cluster 2, promoting more favorable enzyme orientation and substrate accessibility. Conversely, at lower loadings, random orientation involving both clusters could occur, potentially restricting access to the active site and leading to reduced activity, as observed for T@Gx.

Similar to T@Gx, T_HL_@Gx exhibits remarkable thermal stability, maintaining over 100% of its initial activity after 90 min of incubation at 50 °C (Figure 4A). Furthermore, the observed difference in residual activity between T_HL_@Gx and T@Gx can be explained by a protective effect from the more crowded immobilized thrombin population, which enhances resistance to thermal denaturation.^43^ Similar phenomenon was noted with soluble thrombin incubated at 50 °C, where a 10-fold increase in concentration (40 μg·mL^–1^ vs 400 μg·mL^–1^) conferred higher thermal stability (Figure 4A).

To evaluate the reversibility of immobilized thrombin and ensure minimal thrombin leaching during affinity tag digestion, we investigated its stability under aggressive conditions. Specifically, T_HL_@Gx was boiled in Laemmli lysis buffer for 5 min, followed by SDS-PAGE analysis of the supernatant to detect any leached thrombin if immobilization were reversible. The results confirmed robust and irreversible thrombin binding to the AG-Gx support, with negligible thrombin leaching even under extreme boiling conditions (Figure S9, lanes 3 and 4).

Additionally, 6xHis-FNR digested by T_HL_@Gx showed high purity, with no detectable thrombin contamination or undigested 6xHis-FNR (Figure S9, lane 5). Due to the close molecular weights of the thrombin heavy chain (33 kDa) and digested FNR (34.3 kDa), distinguishing between them in the employed SDS-PAGE conditions is challenging. To address this, we conducted a parallel assay digesting the 6xHis- GFP, which has a molecular weight of 29 kDa, while its digested form weighs 27.3 kDa, thus providing a clearer separation from thrombin (Figure S9, lanes 10–12). Freshly prepared T_HL_@Gx exhibited no leaching even after 5 min of boiling in Laemmli lysis buffer (Figure S9, lane 9). Similarly, after a 1-h digestion of 6xHis-GFP, only nonspecifically adsorbed GFP was observed when boiling the used T_HL_@Gx in Laemmli lysis buffer, with no evidence of thrombin contamination (Figure S9, lane 10). The final digested GFP (eluate 2) also demonstrated a high degree of purity, free from thrombin or undigested 6xHis-GFP contamination (Figure S9, lane 11). These findings underscore the remarkable stability and effectiveness of the T_HL_@Gx system in ensuring minimal enzyme leaching and high product purity. From this point onward, T_HL_@Gx was used for all subsequent analyses.

### Affinity Tag Digestion of Different Enzymes and Tags

To explore the utility of the selected heterogeneous thrombin, we evaluated the 6xHis-tag digestion of other enzymes and proteins displaying different oligomeric states, presenting an increased complexity for effective 6xHis-tag cleavage. Specifically, we targeted 6xHis-tagged monomeric FNR and GFP, dimeric FDH, hexameric AlaDH, and octameric GlyDH (Figure 5A).

**Figure 5:**
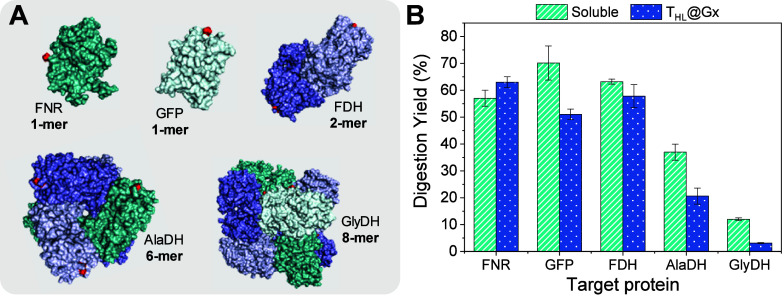
Performance of heterogeneous thrombin in affinity tag digestion of various target enzymes and proteins. (A) Surface representation of target enzymes and proteins: FNR (PDB 1GJR); GFP (PDB 1GFL); FDH (PDB 5DN9); AlaDH (The three-dimensional structure was generated by homology modeling using the SWISS-MODEL platform,^44^ with SMTL ID: 8hyh.1 as the template) and GlyDH (PDB 1JQ5). (B) Digestion yield of 6xHis-tagged target enzymes or proteins (250 μL, 2.0 mg·mL^–1^) incubated with either soluble thrombin or immobilized thrombin (T_HL_@Gx, 5 mg, at 1 mg_thrombin_·g_support_^–1^) at a 100:1 target protein-to-thrombin mass ratio in 50 mM Tris buffer pH 8.0. Reactions were conducted at 4 °C for 1 h with gentle rotation (30 rpm).

After 1 h, both soluble and immobilized thrombin achieved highly similar digestion yields for each target protein, with variations of less than 20% between them (Figure 5B). However, the specific digestion yield varied across the different target proteins, as detailed below. Monomeric and dimeric proteins/enzymes (GFP, FNR and FDH) displayed the highest digestion yields (50–70%), consistent with the greater accessibility of their 6xHis-tags to thrombin cleavage, as these tags are more solvent-exposed (Figures 5B and S10–S12). In contrast, hexameric (6-mer) and octameric (8-mer) enzymes exhibited lower digestion yields, below 40%, due to reduced accessibility of their 6xHis-tags (Figures 5B and S13 and S14). Notably, the octameric GlyDH exhibited the lowest digestion yield, not due to its multimeric nature but rather because its 6xHis-tags are confined to small intersubunit regions, reducing their accessibility to thrombin cleavage (Figure S14).

These digestion profiles highlight the broad applicability of heterogeneous thrombin, which closely mimics the behavior of its soluble form. However, unlike soluble thrombin, the immobilized biocatalyst exhibits exceptional stability during repeated batch digestion cycles, retaining over 100% of its initial activity after 10 reuse cycles (Figure 6A). This remarkable stability allows the immobilized thrombin to achieve an accumulated total turnover number (TTN) approaching 600— significantly surpassing the capacity of its soluble counterpart, which cannot be reused. This performance positions T_HL_@Gx as a promising heterogeneous biocatalyst for integration into large-scale or continuous protein purification workflows, offering enhanced efficiency, reusability, and operational stability. Other study reported a stable immobilized thrombin preparation achieved through immobilization on polyacrylamide gels, where the enzyme retained 75% of its residual activity after 10 cycles of reuse for cleaving the S-thanatin fusion protein.^45^

**Figure 6:**
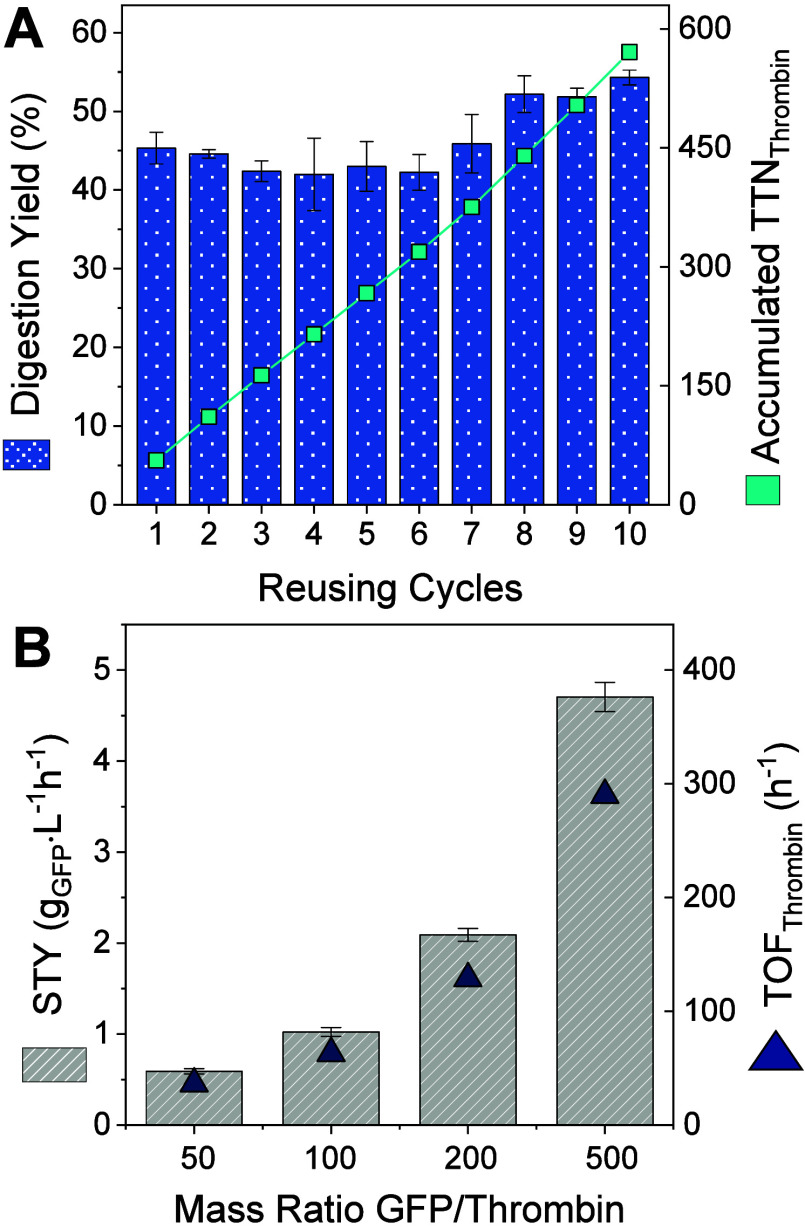
Operational Stability and Process Intensification. (A) Operational stability of T_HL_@Gx in the digestion of 6xHis-GFP as the target protein. Every cycle corresponds to a digestion of a 6xHis-GFP (250 μL, 2 mg·mL^–1^) incubated with 5 mg of T_HL_@Gx at a 100:1 target protein-to-thrombin mass ratio in 50 mM Tris buffer pH 8.0, at 4 °C for 1 h. Total turnover number (TTN) was defined as the ratio of digested 6xHis-GFP (mol) to thrombin used (mol). Each cycle corresponds to 1 h of digestion under identical conditions. (B) Digestion intensification. 6xHis-GFP (250 μL) at varying concentrations (1, 2, 4, or 10 mg·mL^–1^) was incubated with 5 mg of T_HL_@Gx in 50 mM Tris buffer pH 8.0 at 4 °C for 1 h. Space time yield (STY) was defined as the amount of digested 6xHis-GFP (g) per liter per hour. Turnover frequency (TOF) was defined as the moles of digested 6xHis-GFP per mole of thrombin per hour.

To further evaluate the versatility of the T_HL_@Gx biocatalyst, we tested its ability to cleave two additional fusion constructs containing different N-terminal domains. Specifically, we examined the digestion profile of a dimeric reductive aminase (RedAm) featuring an N-terminal Strep-tag separated from the enzyme by a thrombin recognition site (Strep-RedAm) (Figures S15A and S15C). In addition, we assessed a bulkier fusion partner composed of a SpyCatcher (SpyC) protein domain, commonly used in bio-orthogonal protein bioconjugation.^46^ This construct corresponds to a formate dehydrogenase (FDH) enzyme fused to an N-terminal SpyC domain (SpyC-FDH) (Figures S15B and S15D). The digestion profiles demonstrate the broad applicability of heterogeneous thrombin, which closely replicates the behavior of its soluble form, achieving comparable digestion yields. However, due to restricted enzyme accessibility upon immobilization, longer incubation times are required (Figure S16 and S17). Specifically, complete digestion of Strep-RedAm and SpyC-FDH by soluble thrombin was achieved within 2 and 4 h, respectively. Prolonged incubation beyond these time points resulted in nonspecific and undesired degradation products (Figure S16, lanes 6–11). In contrast, immobilized thrombin achieved similar product yields after 24 h of incubation at 4 °C, producing highly pure products (Figure S16, lanes 13–18; Figure S17, lane 7). These findings underscore that even challenging or sterically hindered affinity tags can be efficiently cleaved by optimizing incubation times, enabling the production of highly pure target proteins.

Additionally, to assess its economic feasibility, we challenged the heterogeneous thrombin to higher target protein concentrations to determine its productivity limit. To this aim, we scaled up from 50:1 to 500:1 protein-to-thrombin mass ratio, corresponding to initial target 6xHis-GFP concentrations of 0.5 to 10 mg·mL^–1^. Within this target protein concentration range, the heterogeneous thrombin demonstrates a proportional increase in substrate-to-thrombin yield with increasing initial target protein concentration, achieving space-time yields (STY) of up to 4.7 g·L^–1^·h^–1^ for digested GFP (Figure 6B). The STY, defined as the amount of product (mP) formed per unit of residence time (τ) and reaction volume (V) [STY = mP·(τ·V)^−1^], is a key metric to evaluate biocatalyst performance. While highly productive, large-scale biocatalytic processes often require STY values above 100 g·L^–1^·h^–1^,^47^ for high-value products exceeding $100·kg^–1^, STY values between 1 and 10 g·L^–1^·h^–1^ are generally considered sufficient for economic viability.^48^ To the best of our knowledge, the STY obtained with our system represents the highest reported for affinity tag digestion, further supporting the industrial potential of the T_HL_@Gx biocatalyst. These results reflect the economic advantage and scalability potential of the immobilized thrombin system developed in this work.

Finally, we benchmarked the performance of our T_HL_@Gx biocatalyst against a commercially available thrombin preparation kit to highlight its advantages in catalytic efficiency, stability, and cost-effectiveness. For a fair and direct comparison, both affinity tag digestion and thermal stability assays were carried out under identical reaction conditions to those used for the T_HL_@Gx system. Notably, the Novagen kit employs a soluble biotinylated thrombin that requires removal via a secondary step using streptavidin-functionalized agarose. To align the formats for comparison, we immobilized the kit’s biotinylated thrombin onto the provided streptavidin-agarose beads, matching the enzyme loading of the T_HL_@Gx system (1.0 mg·g^–1^). Notably, the kit’s manufacturer-recommended digestion protocol requires incubation for up to 16 h, significantly longer than the 1-h reaction time used in our assays. Furthermore, the immobilized thrombin preparation achieved highly stable, irreversible binding, as evidenced by its resistance to elution even after boiling in Laemmli lysis buffer for 5 min (Figure S18), underscoring the robustness of this immobilization strategy.

Under these similar working conditions, T_HL_@Gx outperformed the commercial preparation, achieving up to 1.7-fold higher digestion yields and maintaining 1.5-fold higher residual activity after thermal treatment at 50 °C (Table 2). Beyond its superior catalytic and stability profiles, the T_HL_@Gx was also nearly 10 times more cost-effective than the commercial kit (Table 2). We also aimed to compare T_HL_@Gx with a covalently immobilized thrombin preparation available from Sigma-Aldrich. However, this comparison could not be performed due to the product’s unavailability, as it remained on backorder for over five months without delivery, underscoring its limited accessibility. Collectively, these results highlight the enhanced functional robustness, scalability, and economic viability of the T_HL_@Gx system, positioning it as a compelling alternative for affinity tag removal in both research and industrial settings.

**Table 2:** Performance of Commercial Immobilized Thrombin Preparations

Brand	Price (€·mL^–1^)	Digestion yield (%)^a^	Thermal stability (%)^b^
Novagen	1367	38 ± 3	82 ± 2
This work	150	64 ± 2	124 ± 4

a Affinity tag digestion was performed by incubating 6xHis-FNR (0.2 mg·mL^–1^) with thrombin biocatalysts at a protein-to-thrombin mass ratio of 100:1 in 50 mM Tris buffer (pH 8.0). The reaction was carried out at 4 °C with gentle agitation (30 rpm) for 1 h, using a support-to-FNR mass ratio of 1:50 (w/w).

b Thermal stability is expressed as the residual activity of the biocatalyst following 1.5 h of incubation at 50 °C in 50 mM Tris buffer pH 8.0.

## Conclusions

In this study, we developed a highly stable and reusable heterogeneous thrombin biocatalyst for efficient affinity tag digestion. Leveraging the Python-based application CapiPy, we analyzed the thrombin surface to identify key residues suitable for immobilization, optimizing its performance on aldehyde- and epoxide-functionalized agarose supports. Thrombin immobilized on glyoxyl-activated agarose microbeads demonstrated superior performance, achieving 6xHis-tag digestion rates comparable to its soluble counterpart, while enabling seamless reusability and straightforward separation from the digested target protein. The immobilized thrombin exhibited remarkable thermal and operational stability, maintaining catalytic efficiency across a wide target protein-to-thrombin mass ratio range of 50:1 to 500:1, producing up to 4.7 g·L^–1^·h^–1^ of digested protein. Its robust performance and adaptability make it suitable for processing various multimeric enzymes, diverse peptide tags, and other fusion proteins, further highlighting its versatility. These findings underscore the potential of immobilized thrombin in enhancing recombinant protein purification workflows, offering scalable and cost-effective solutions for industrial and research applications. Its stability, reusability, and ease of integration into existing purification platforms are particularly valuable for large-scale manufacturing, where consistent performance and minimal enzyme loss are critical. Additionally, its applicability extends to various biotechnological and pharmaceutical sectors, including therapeutic protein production, vaccine development, and diagnostic reagent preparation. Looking ahead, future advancements in immobilization technologies and support materials could further improve its compatibility with continuous processing systems, reinforcing its role as a key biocatalyst in next-generation protein purification processes.

## Supplementary Material



## Data Availability

The PDB file used in this study is available for download.

## Notes

Schemes that include a spectrofluorometric assay for measuring thrombin activity and a palette of functionalized solid microporous agarose beads; additional supporting figures featuring SDS–PAGE analysis of various commercial thrombin extracts, the FASTA sequence of the PDB file used for thrombin analysis, thrombin amino acid dynamics, thrombin immobilization kinetics, and structural analysis of surface-exposed negatively charged clusters in thrombin; the thermal denaturation curve of soluble thrombin, intrinsic fluorescence analyses of thrombin-based biocatalysts, and an *in silico* study on thrombin orientation via immobilization through both lysine clusters; SDS–PAGE analyses for thrombin leaching assessments, as well as for Strep-RedAm and SpyC-FDH following IMAC purification and affinity tag digestion; surface representations of FNR, GFP, FDH, AlaDH, GlyDH, Strep-RedAm and SpyC-FDH included to complement the study’s findings (PDF)

## Abbreviations

AlaDH: l-alanine dehydrogenase
AMC: 7-amino-4-methylcumarin
AG: 4% cross-linked agarose beads
AG-A/G: amino-glutaraldehyde functionalized AG
AG-E: epoxy activated AG
AG-Gx: glyoxyl activated AG
AG-IDA/E: AG functionalized with epoxide and IDA groups
Boc-VPR-AMC: Boc-Val-Pro-Arg-7-amido-4-methylcoumarin hydrochloride
E: epoxy groups
EDA: ethylenediamine
FDH: formate dehydrogenase
FNR: ferredoxin NADPH reductase
GA: glutaraldehyde
GFP: green fluorescent protein
GlyDH: glycerol dehydrogenase
IDA: iminodiacetic acid
IMAC: immobilized metal affinity chromatography
IPTG: isopropyl-β-d-thiogalactopyranoside
RedAm: reductive aminase
RMSD: root-mean-square deviation
SASA: solvent-accessible surface area
SpyC-FDH: SpyCatcher fused to FDH
Strep-RedAm: Strep-tagged RedAm
STY: space time yield
T@A/G: thrombin immobilized on AG-A/E
T@E: thrombin immobilized on AG-E
T@Gx: thrombin immobilized on AG-Gx at a load of 0.1 mg·g^–1^
T_HL_@Gx: thrombin immobilized on AG-Gx at a load of 1 mg·g^–1^
TOF: turnover frequency
TTN: total turnover number
