# Supplementary material for: Immobilization of Thrombin on Agarose-Based Supports for Affinity Tag Removal
Source: Biomacromolecules. 2025 Jul 1;26(7):4718–29. doi: 10.1021/acs.biomac.5c01010 (PMC12264962; doi:10.1021/acs.biomac.5c01010)
Supplement: Supplementary file 1 [file bm5c01010_si_001.pdf]

Supporting Information

Affinity tag removal by immobilized thrombin on agarose-based supports

Juan Cruz Almada,[a] *Miguel Marco-Martin,*[a] *David Roura-Padrosa,*[b] *and Susana Velasco-Lozano*[a,c]*

[a] Instituto de Síntesis Química y Catálisis Homogénea (ISQCH), CSIC-Universidad de Zaragoza, C/ Pedro Cerbuna, 12, 50009, Zaragoza, Spain.
[b] inSEIT AG, Gesellschaftsstrasse 42, 3012 Bern, Switzerland.
[c] Aragonese Foundation for Research and Development (ARAID), Av. Ranillas 1-D, 50018, Zaragoza, Spain.

*Corresponding author: S. Velasco-Lozano
Phone: +34 976976842271, Ext. 842271
E-mail address: svelasco@unizar.es

Table of contents

Supporting Schemes S3

Scheme S1. Spectrofluorometric assay for thrombin activity measurement S3

Scheme S2. Palette of functionalized solid microporous agarose beads S3

Supporting Figures S4

Figure S1. The SDS-PAGE of the different commercial thrombin preparations S4

Figure S2. FASTA sequence of the PDB file used for thrombin analysis. S5

Figure S3. Thrombin amino acid dynamics. S6

Figure S4. Thrombin immobilization kinetics S7

Figure S5. Structural analysis of surface-exposed negatively charged clusters in thrombin S8

Figure S6. Thermal denaturation curve of soluble thrombin. S9

Figure S7. Intrinsic fluorescence analysis of thrombin biocatalysts. S10

Figure S8. *In silico* analysis of thrombin orientation upon immobilization via lysine clusters 1 and 2. .... S11

Figure S9. SDS-PAGE analysis of thrombin leaching assessment. S12

Figure S10. Surface representation of ferredoxin $NADP^+$ reductase S13

Figure S11. Surface representation of green fluorescent protein S14

Figure S12. Surface representation of formate dehydrogenase S15

Figure S13. Surface representation of alanine dehydrogenase S16

Figure S14. Surface representation of glycerol dehydrogenase S17

Figure S15. Surface representation of Strep-RedAm and SpyC-FDH constructs S18

Figure S16. SDS-PAGE analysis of Strep-RedAm following IMAC purification anSd affinity tag digestion S19

Figure S17. SDS-PAGE analysis of SpyC-FDH following IMAC purification and affinity tag digestion S20

Figure S18. SDS-PAGE analysis assessing the leaching of commercial biotinylated thrombin immobilized on streptavidin-agarose beads. S21

Supporting References S22

Supporting Schemes

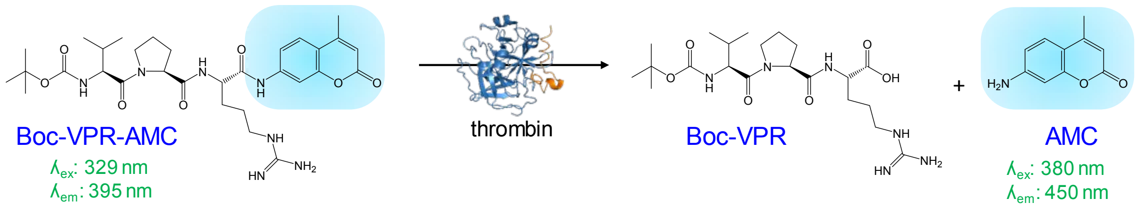

Scheme S1. Spectrofluorometric assay for thrombin activity measurement.

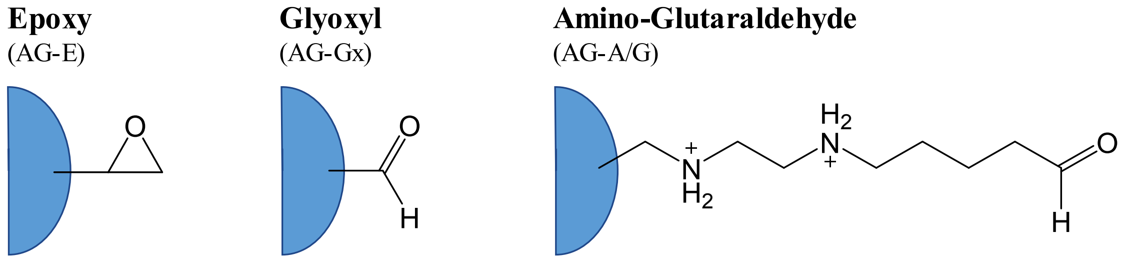

Scheme S2. Palette of functionalized solid microporous agarose beads screened for thrombin immobilization.

Supporting Figures

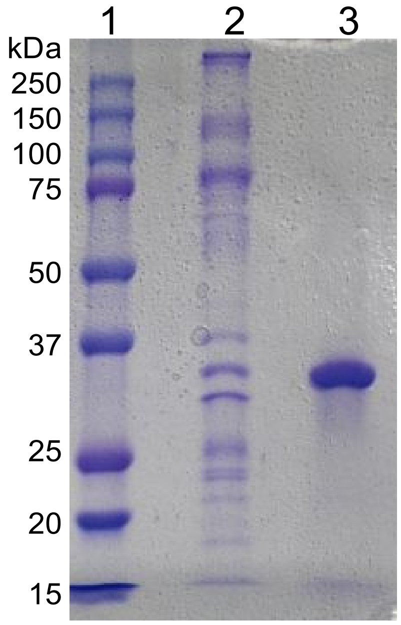

Figure S1. The SDS-PAGE of the different commercial thrombin preparations. Lanes: 1: Molecular weight marker (BioRad Precision Plus Protein All Blue Standard). 2: Commercial thrombin cat. T7326. 3: Commercial thrombin cat. 605157.

Thrombin Sequence in the Used PDB File

>Thrombin_LightChain

EADCGLRPLFEKKQVQDQTEKELFESYIE

>Thrombin_HeavyChain

IVEGQDAEVGLSPWQVMLFRKSPQELLCGASLISDRWVLTAAHCLLYPPWDKN

FTVDDLLVRIGKSRTRYERKVEKISMLDKIYIHPRYNWKENLDRDIALLKLKRPI

ELSDYIHPVCLPDKQTAAKLLHAGFKGRVTGWGNRREVAEVQPSVLQVVNLPL

VERPVCKASTRIRITDNMFCAGYKPGEGKRGDACEGDSGGPFVMKSPYNNRW

YQMGIVSWGEGCDRDGKYGFYTHVFRLKKWIQKVIDRLGS

Sequence alignment:

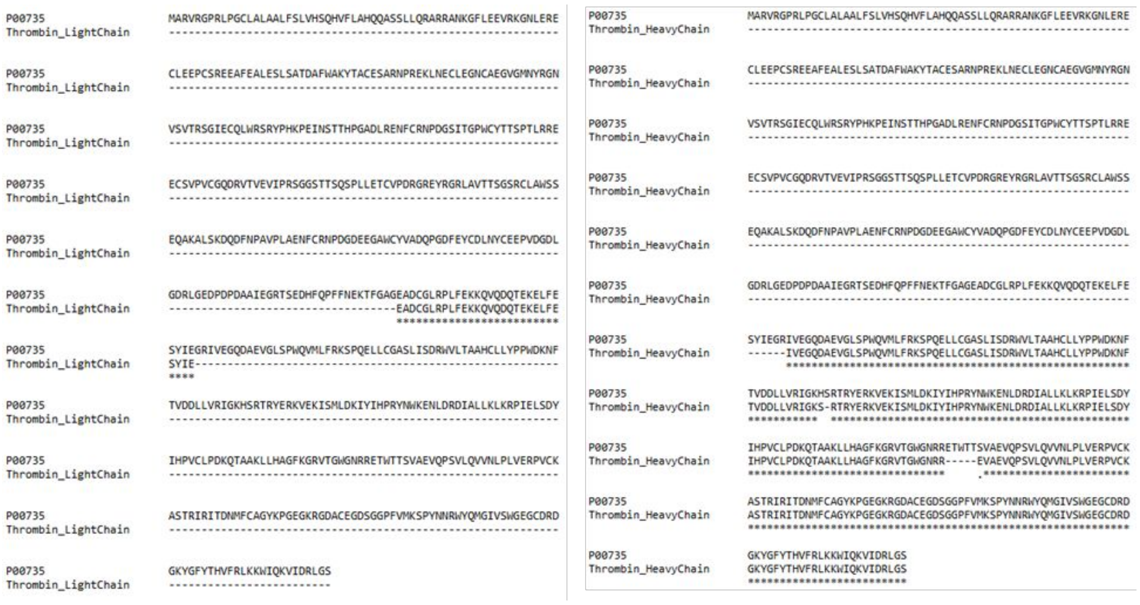

Figure S2. FASTA sequence of the PDB file used for thrombin analysis. An alignment between the light and heavy chain of the PDB compared to the pre-thrombin found in Uniprot (ID: P00735) is also attached.

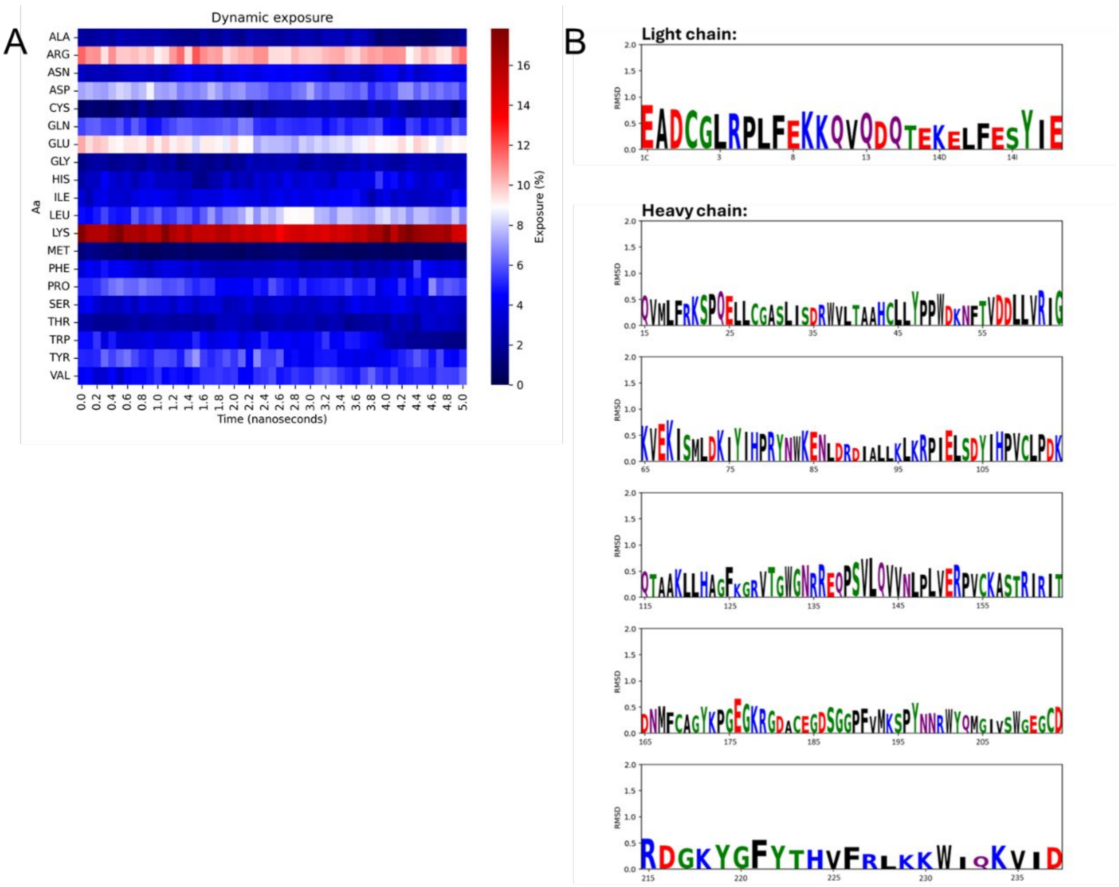

Figure S3. Thrombin amino acid dynamics. **A.** Heatmap of dynamic surface amino acid exposure during the simulation, with warmer colors reflecting higher levels of exposure. **B.** Residue-specific RMSD logo representation. Amino acids are color-coded following the WebLogo Chemistry scheme: Red for D, E; Green for G, S, T, Y, C; Blue for H, K, R; Purple for N, Q; and Black for all other residues. Note that in this figure all residue numbers correspond to the modified PDB version (see Materials and Methods, and Figure S2 for details).

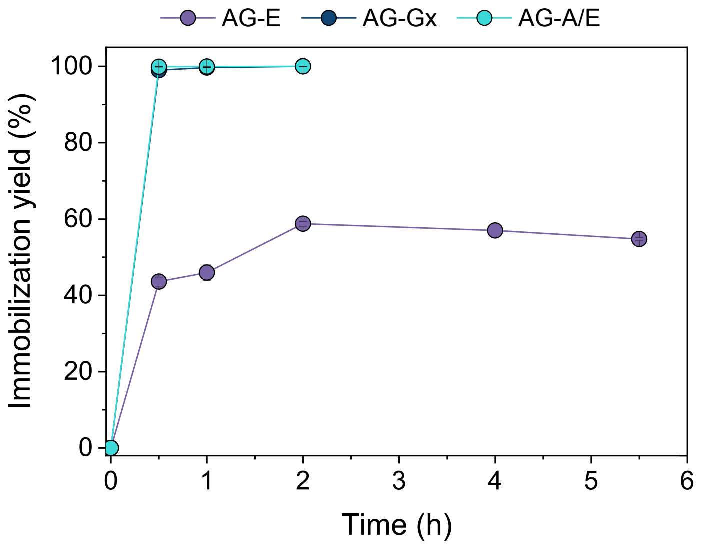

Figure S4. Thrombin immobilization kinetics on functionalized agarose microbeads. In all cases, the immobilization was performed at 4 ºC, with an initial offered thrombin load of 0.1 $mg \cdot g_{support}^{-1}$. In all cases, a control solution of soluble thrombin in the respective buffer was incubated under the same conditions, without support, to assess enzyme stability at the specific pH levels. All control samples retained more than 98% of their respective initial activity under these conditions.

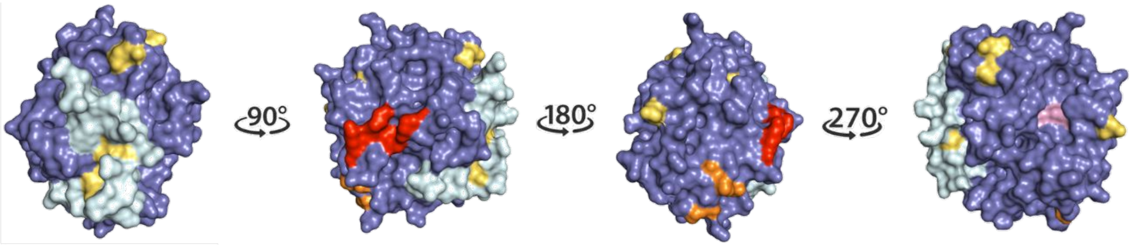

Figure S5. Structural analysis of surface-exposed negatively charged clusters in thrombin (PDB 3PMB). Negatively charged Cluster 1 (E11, D17 and E20), Cluster 2 (D74, D76, D151), and Cluster 3 (E162, D200 and D202), all are highlighted in yellow. Surface-exposed lysine clusters: Cluster 1 (K60, K81, K83) is highlighted in orange, while Cluster 2 (K100, K215, K216, K220) is shown in red. The heavy chain is depicted in slate blue, and the light chain in pale cyan. The catalytic triad residues (S173, H29, D76) are marked in pink. Note that in this figure all residue numbers correspond to the modified PDB version (see Materials and Methods, and Figure S2 for details).

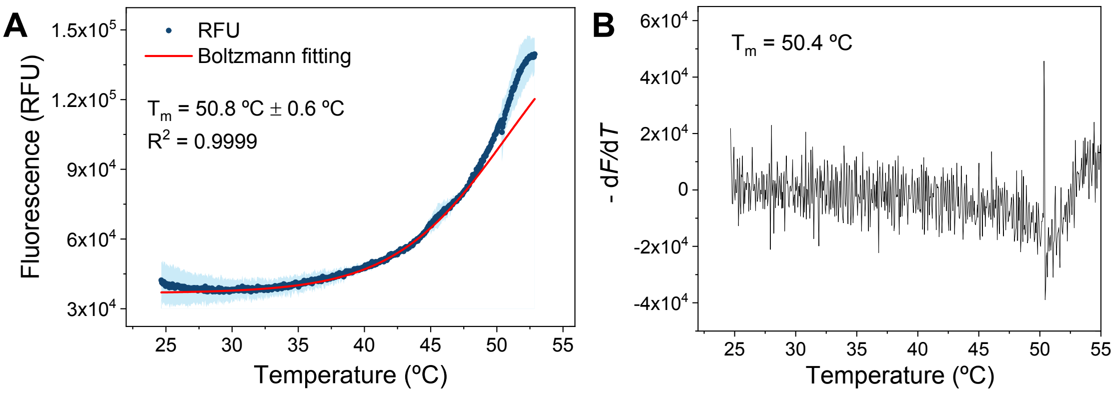

Figure S6. Thermal denaturation curve of soluble thrombin. The melting temperature (T_m) was determined using two approaches: **A**. non-linear Boltzmann fitting of the fluorescence emission data, with the standard deviation of three independent replicates represented as a light blue shaded area. **B**. Analysis of the first derivative of fluorescence emission with respect to temperature ($-dF/dT$).[S1]

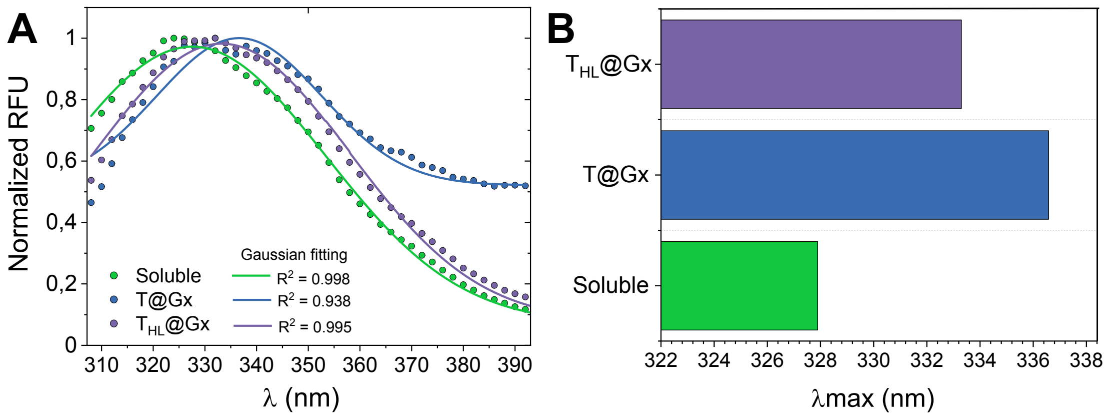

Figure S7. Intrinsic fluorescence analysis of thrombin biocatalysts. **A**. Normalized fluorescence emission spectra with Gaussian fitting curves. The relative fluorescence units (RFUs) of each sample were normalized to their respective λmax, setting the intensity at λmax to a value of 1. **B**. Comparison of maximum emission wavelengths.

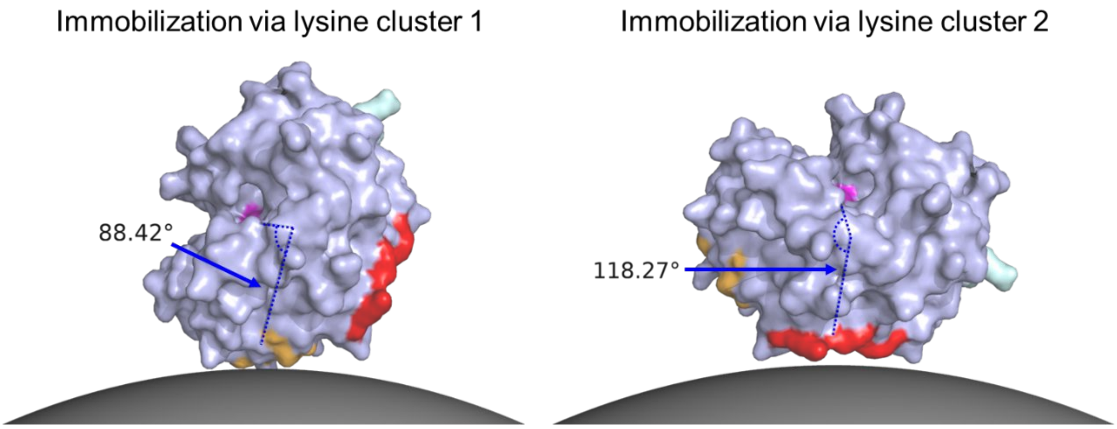

Figure S8. *In silico* analysis of thrombin orientation upon immobilization via lysine clusters 1 and 2. Lysine residues from cluster 1 and cluster 2 are shown in orange and red, respectively. Orientation angles were calculated from the thrombin active site (pink) to the most distal atom within each lysine cluster, offering insights into the enzyme's spatial configuration upon attachment.

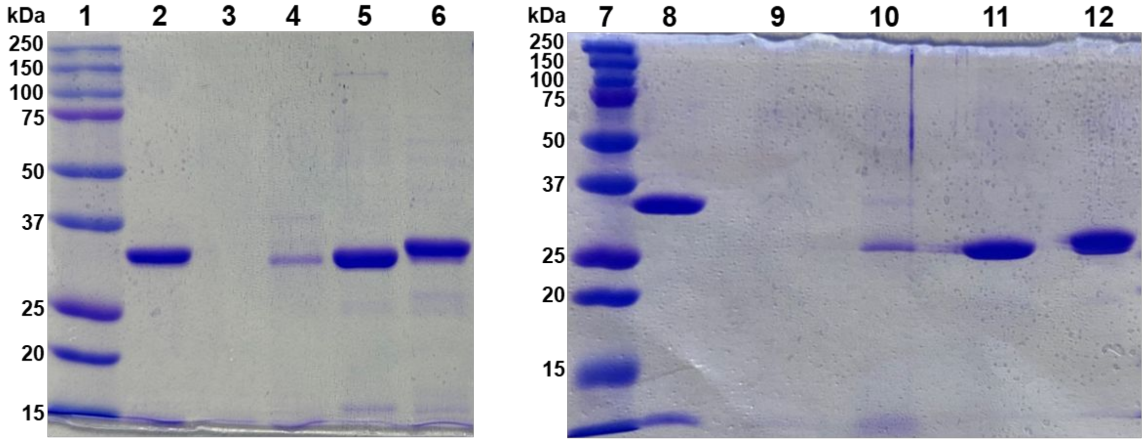

Figure S9. SDS-PAGE analysis of thrombin leaching assessment.
Lanes:

1. Molecular weight marker (Bio-Rad Precision Plus Protein All Blue Standard).
2. Soluble thrombin (heavy chain, 33 kDa) at a concentration corresponding to the total theoretical thrombin leaching from T_{HL}@Gx (100%).
3. Supernatant of freshly prepared T_{HL}@Gx after boiling with Laemmli lysis buffer.
4. Supernatant obtained after boiling the used T_{HL}@Gx (following a 1-hour digestion of 6xHis-FNR) in Laemmli lysis buffer.
5. Digested FNR (34.4 kDa) from eluate 2.
6. Undigested 6xHis-FNR (36.5 kDa).
7. Molecular weight marker (Bio-Rad Precision Plus Protein All Blue Standard).
8. Soluble thrombin (heavy chain, 33 kDa) at a concentration equivalent to the total theoretical thrombin leaching from T_{HL}@Gx (100%).
9. Supernatant of freshly prepared T_{HL}@Gx after boiling with Laemmli lysis buffer.
10. Supernatant obtained after boiling the used T_{HL}@Gx (following a 1-hour digestion of 6xHis-GFP) in Laemmli lysis buffer.
11. Digested GFP (27.3 kDa) from eluate 2.
12. Undigested 6xHis-GFP (29.0 kDa).

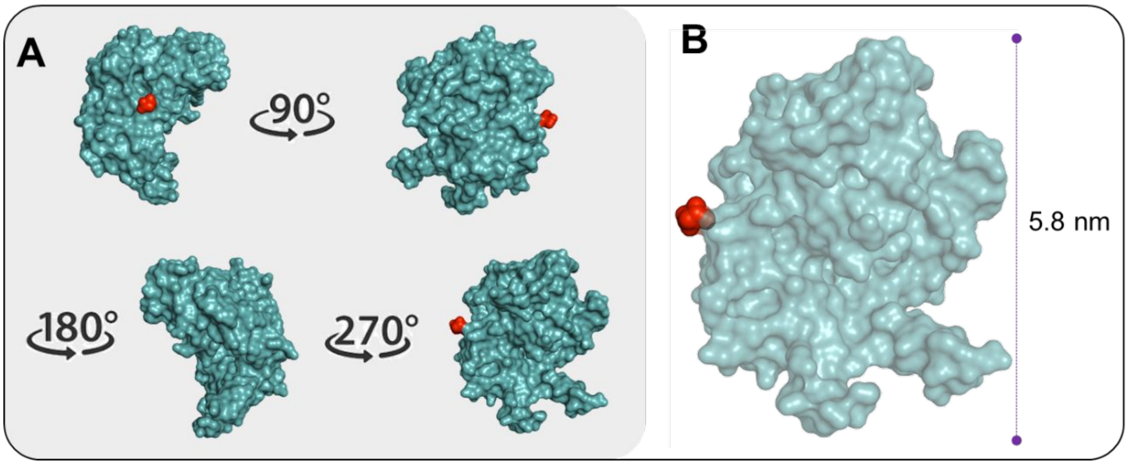

Figure S10. Surface representation of ferredoxin $NADP^+$ reductase, FNR (PDB 1GJR). **A.** A three-dimensional rotational view of the monomeric conformation. **B.** Transparent surface representation of the FNR monomer. In both panels, the monomer is colored in light teal cyan. The N-terminus, where the 6xHis-tag is bound, is depicted in red spheres, emphasizing the location of these tag on the surface of the monomer.

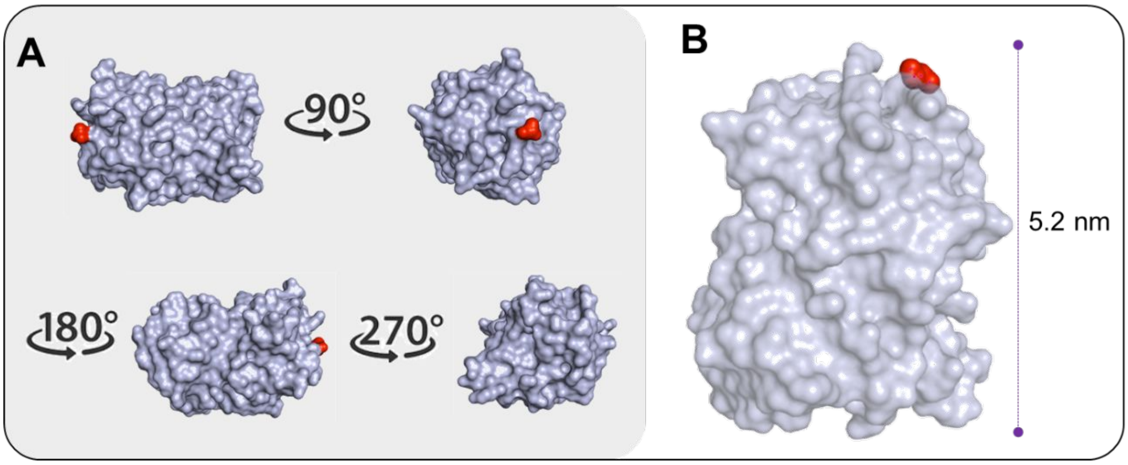

Figure S11. Surface representation of green fluorescent protein, GFP (PDB 1GFL). **A.** A three-dimensional rotational view of the monomeric conformation. **B.** Transparent surface representation of the GFP monomer. In both panels, the monomer is colored in light blue. The N-terminus, where the 6xHis-tag is bound, is depicted in red spheres, emphasizing the location of these tag on the surface of the monomer.

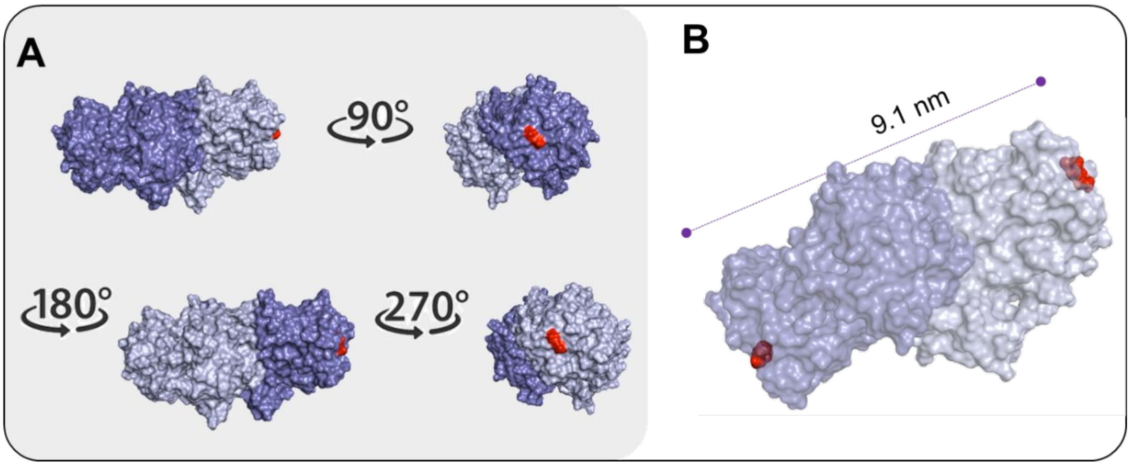

Figure S12. Surface representation of formate dehydrogenase, FDH (PDB 5DN9). **A.** A three-dimensional rotational view of the dimeric conformation, this view showcases the spatial arrangement between the two monomers that constitute the dimer. **B.** Transparent surface representation of the FDH dimer. In both panels, each of the two monomers is distinctly colored: in slate blue and in light blue, allowing for clear visualization of the dimeric structure. The N-terminus, where the 6xHis-tag is bound, is depicted in red spheres, emphasizing the location of these tags on the surface of the dimer.

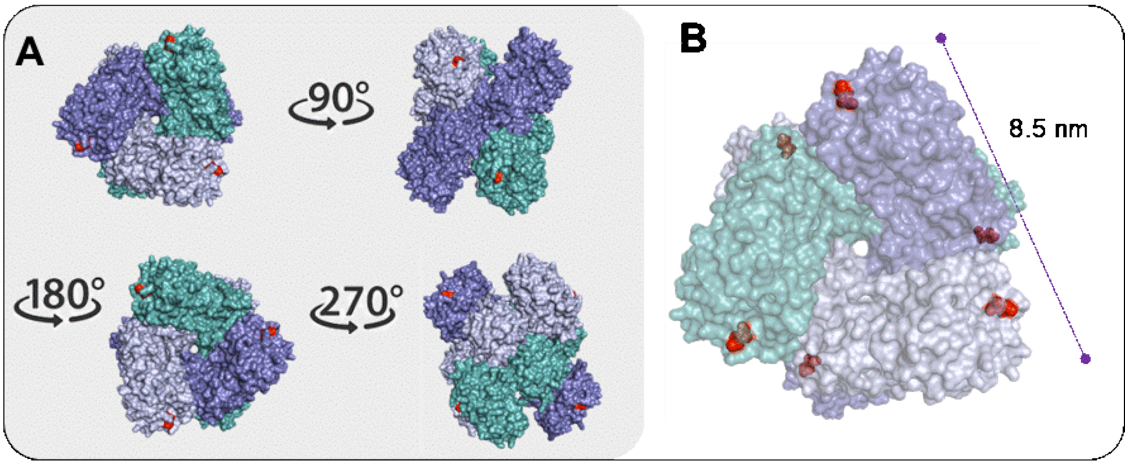

Figure S13. Surface representation of alanine dehydrogenase, AlaDH. The three-dimensional structure was generated by homology modeling using the SWISS-MODEL platform,[S2] with SMTL ID: 8hyh.1 as the template. **A.** A three-dimensional rotational view of the hexameric conformation, this view showcases the spatial arrangement between the six monomers that constitute the hexamer. **B.** Transparent surface representation of the AlaDH hexamer. In both panels, each of the six monomers is distinctly colored: two in slate blue, two in light blue, and two in light teal cyan, allowing for clear visualization of the hexameric structure and symmetry. The N-terminus, where the 6xHis-tag is bound, is depicted in red spheres, emphasizing the location of these tags on the surface of the hexamer.

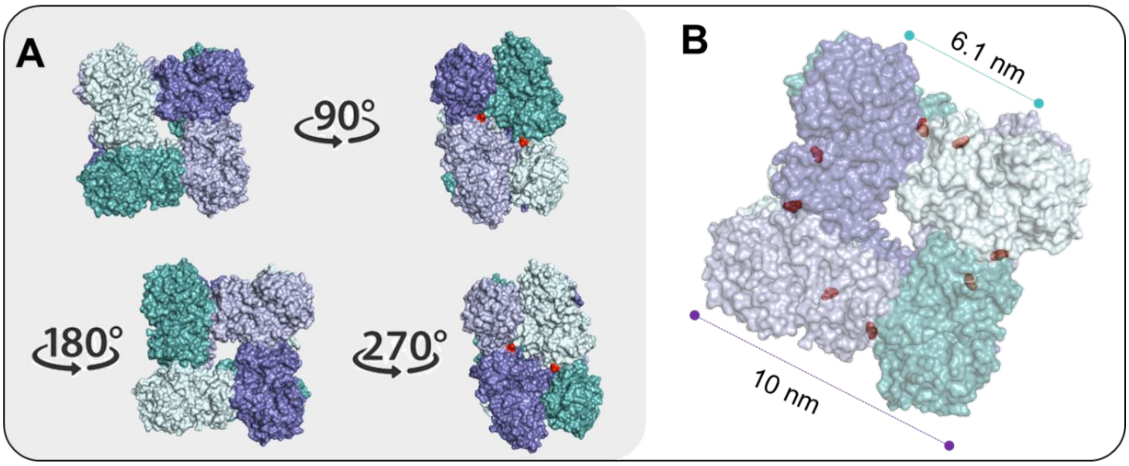

Figure S14. Surface representation of glycerol dehydrogenase, GlyDH (PDB 1JQ5). **A.** A three-dimensional rotational view of the octameric conformation, this view showcases the spatial arrangement between the eight monomers that constitute the octamer. **B.** Transparent surface representation of the GlyDH octamer. In both panels, each of the eight monomers is distinctly colored: two in slate blue, two in light blue, two in light teal cyan, and two in pale cyan, allowing for clear visualization of the octameric structure and symmetry. The N-terminus, where the 6xHis-tag is bound, is depicted in red spheres, emphasizing the location of these tags on the surface of the octamer.

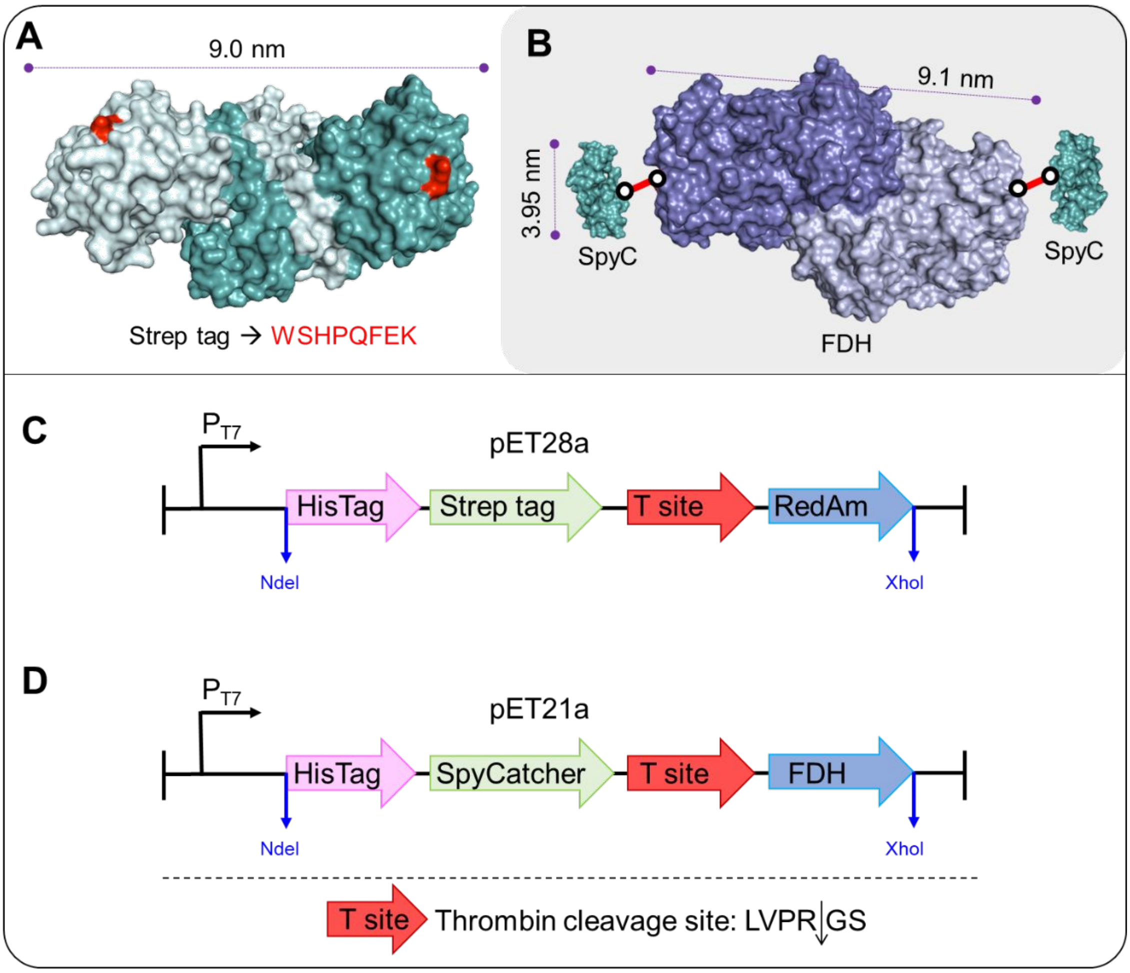

Figure S15. Surface representation of Strep-RedAm and SpyC-FDH constructs. **A**. Structure of Strep-RedAm (PDB: 9FM7), with the N-terminal affinity tag highlighted in red spheres. **B**. Model of SpyC-FDH, where the N-terminal SpyCatcher (SpyC) domain is depicted as red lines. The FDH domain was modeled using PDB: 5DN9, and the SpyC domain from PDB: 4MLI. Both constructs include an N-terminal 6xHis-tag upstream of the Strep-tag or SpyC domain to enable IMAC purification, as well as a thrombin recognition site between the enzyme and the strep-tag or SpyC domains. **C**. Plasmid map of the Strep-RedAm construct. **D**. Plasmid map of the SpyC-FDH construct.

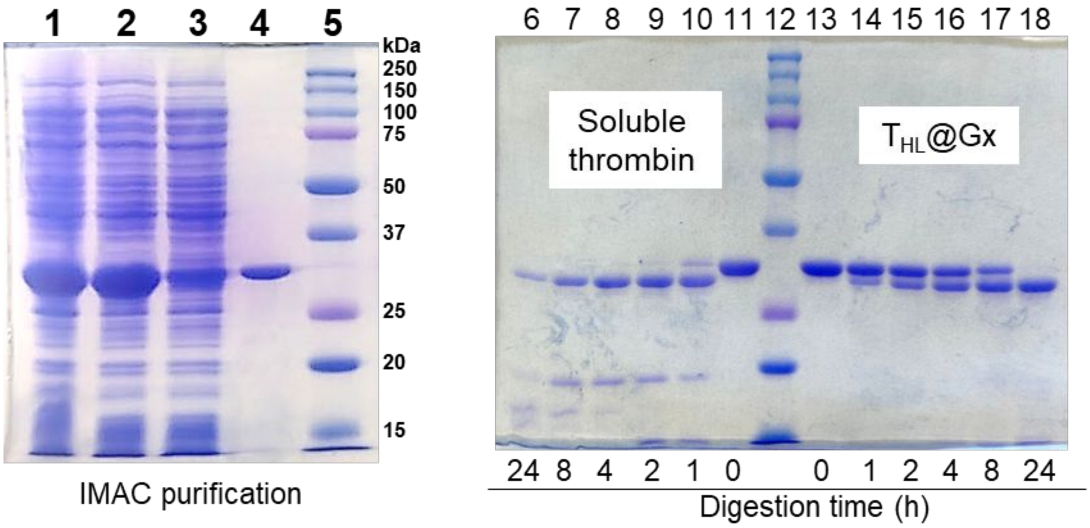

Figure S16. SDS-PAGE analysis of Strep-RedAm following IMAC purification and affinity tag digestion. Digestion conditions: Strep-RedAm (250 µL, 2.0 mg·mL^{-1}) incubated with either soluble or immobilized thrombin (T_{HL}@Gx, 5 mg, at 1 $mg_{thrombin}\cdot g_{support}^{-1}$) at a 100:1 target protein-to-thrombin mass ratio in 50 mM Tris buffer, pH 8.0. Reactions were performed at 4 °C for 1, 2, 4, 8 or 24 hours under gentle rotation (30 rpm).

Lanes:

Strep-RedAm IMAC purification

1: Total crude cell extract

2: Soluble protein fraction

3: IMAC purification flow-through

4: Purified Strep-RedAm (34 kDa)

5: Molecular weight marker (Bio-Rad Precision Plus Protein All Blue Standard).

Digestion kinetics of Strep-RedAm

6-11: Time-course of digestion using soluble thrombin at 24, 8, 4, 2, 1 and 0 hours, respectively.

12: Molecular weight marker (Bio-Rad Precision Plus Protein All Blue Standard).

13-18: Time-course of digestion using T_{HL}@Gx at 0, 1, 2, 4, 8 and 24 hours, respectively.

The expected molecular weight of the digested RedAm fragment is 31 kDa.

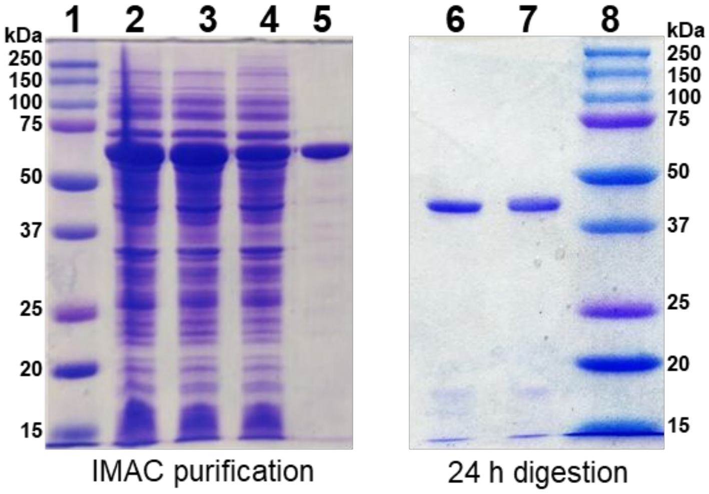

Figure S17. SDS-PAGE analysis of SpyC-FDH following IMAC purification and affinity tag digestion. Digestion conditions: SpyC-FDH (250 µL, 2.0 mg·mL^{-1}) incubated with either soluble or immobilized thrombin (T_{HL}@Gx, 5 mg, at 1 $mg_{thrombin}·g_{support}^{-1}$) at a 100:1 target protein-to-thrombin mass ratio in 50 mM Tris buffer, pH 8.0. Reactions were performed at 4 °C for 24 hours under gentle rotation (30 rpm).

Lanes:

SpyC-FDH IMAC purification

1: Molecular weight marker (Bio-Rad Precision Plus Protein All Blue Standard).

2: Total crude cell extract

3: Soluble protein fraction

4: IMAC purification flow-through

5: Purified SpyC-FDH (56 kDa)

Digestion of SpyC-FDH

6: Digestion product using soluble thrombin corresponding to 24 hours.

7: Digestion product using T_{HL}@Gx corresponding to 24 hours.

The expected molecular weight of the digested FDH fragment is 41 kDa.

8: Molecular weight marker (Bio-Rad Precision Plus Protein All Blue Standard).

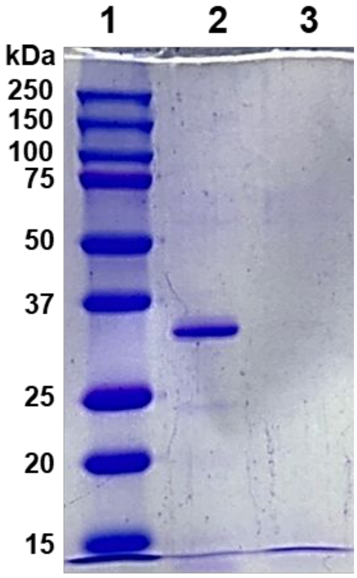

Figure S18. SDS-PAGE analysis assessing the leaching of commercial biotinylated thrombin immobilized on streptavidin-agarose beads.

Lanes:

1: Molecular weight marker (Bio-Rad Precision Plus Protein™ All Blue Standard).

2: Soluble biotinylated thrombin at a concentration equivalent to 100% theoretical leaching from the immobilized form.

3: Supernatant from freshly prepared streptavidin-agarose–immobilized biotinylated thrombin after boiling in Laemmli lysis buffer.

Supporting References

S1. Huynh, K.; Partch, C. L., Analysis of Protein Stability and Ligand Interactions by Thermal Shift Assay. *Curr. Protoc. Protein Sci.* **2015,** *79* (1), 28.9.1-28.9.14.

S2. Waterhouse, A.; Bertoni, M.; Bienert, S.; Studer, G.; Tauriello, G.; Gumienny, R.; Heer, F. T.; de Beer, T. A P.; Rempfer, C.; Bordoli, L.; Lepore, R.; Schwede, T., SWISS-MODEL: homology modelling of protein structures and complexes. *Nucleic Acids Res.* **2018,** *46* (W1), W296-W303.
